# Association between CNS-active drugs and risk of Alzheimer’s and age-related neurodegenerative diseases

**DOI:** 10.3389/fpsyt.2024.1358568

**Published:** 2024-02-29

**Authors:** Helena Cortes-Flores, Georgina Torrandell-Haro, Roberta Diaz Brinton

**Affiliations:** ^1^ Center for Innovation in Brain Science, University of Arizona, Tucson, AZ, United States; ^2^ Department of Pharmacology, University of Arizona College of Medicine, Tucson, AZ, United States; ^3^ Department of Neurology, University of Arizona College of Medicine, Tucson, AZ, United States

**Keywords:** CNS-active drugs, Alzheimer’s disease, retrospective analysis, neurodegenerative diseases, responder analysis

## Abstract

**Objective:**

As neuropsychiatric conditions can increase the risk of age-related neurodegenerative diseases (NDDs), the impact of CNS-active drugs on the risk of developing Alzheimer’s Disease (AD), non-AD dementia, Multiple Sclerosis (MS), Parkinson’s Disease (PD) and Amyotrophic Lateral Sclerosis (ALS) was investigated.

**Research design and methods:**

A retrospective cohort analysis of a medical claims dataset over a 10 year span was conducted in patients aged 60 years or older. Participants were propensity score matched for comorbidity severity and demographic parameters. Relative risk (RR) ratios and 95% confidence intervals (CI) were determined for age-related NDDs. Cumulative hazard ratios and treatment duration were determined to assess the association between CNS-active drugs and NDDs at different ages and treatment duration intervals.

**Results:**

In 309,128 patients who met inclusion criteria, exposure to CNS-active drugs was associated with a decreased risk of AD (0.86% vs 1.73%, RR: 0.50; 95% CI: 0.47-0.53; p <.0001) and all NDDs (3.13% vs 5.76%, RR: 0.54; 95% CI: 0.53-0.56; p <.0001). Analysis of impact of drug class on risk of AD indicated that antidepressant, sedative, anticonvulsant, and stimulant medications were associated with significantly reduced risk of AD whereas atypical antipsychotics were associated with increased AD risk. The greatest risk reduction for AD and NDDs occurred in patients aged 70 years or older with a protective effect only in patients with long-term therapy (>3 years). Furthermore, responders to these therapeutics were characterized by diagnosed obesity and higher prescriptions of anti-inflammatory drugs and menopausal hormonal therapy, compared to patients with a diagnosis of AD (non-responders). Addition of a second CNS-active drug was associated with greater reduction in AD risk compared to monotherapy, with the combination of a Z-drug and an SNRI associated with greatest AD risk reduction.

**Conclusion:**

Collectively, these findings indicate that CNS-active drugs were associated with reduced risk of developing AD and other age-related NDDs. The exception was atypical antipsychotics, which increased risk. Potential use of combination therapy with atypical antipsychotics could mitigate the risk conferred by these drugs. Evidence from these analyses advance precision prevention strategies to reduce the risk of age-related NDDs in persons with neuropsychiatric disorders.

## 1 Introduction

Neuropsychiatric disorders, while common among age-associated neurodegenerative diseases (NDDs), can be a harbinger of the disease and emerge during the prodromal phase of Alzheimer’s disease (AD) (1–3), Parkinson’s disease (PD) (4, 5), Multiple Sclerosis (MS) (6, 7) and Amyotrophic Lateral Sclerosis (ALS) (8). Their emergence during the prodromal phase is suggestive of shared pathological drivers of neuropsychiatric and neurodegenerative diseases.

Neuropsychiatric disorders share common features of NDDs, particularly with AD, including increased oxidative stress, inflammation, disruption of the excitatory/inhibitory balance, and protein aggregation (9–21). Chief among these features are beta amyloid deposition and tau hyperphosphorylation and accumulation, which have been observed in depression, insomnia, and epilepsy (13, 22–26).

Signature pathological hallmarks of AD are seen in depression patients including beta amyloid deposition, tau accumulation, chronic inflammation, and deficits in nerve growth factors, particularly brain-derived neurotrophic factor (BDNF) (22, 23, 14, 11, 16, 27, 28). The serotonergic system, implicated in both conditions, influences non-amyloidogenic amyloid precursor protein (APP) release and neuroplasticity processes, while disruption of the noradrenergic system affects inflammation, amyloid deposition, and neuroprotection (29, 30, 31, 27, 32, 33). Long-term poor sleep quality and AD share common features including chronic inflammation, a reduction of neurotrophic factors including BDNF, alterations in blood-brain barrier (BBB) permeability, and accumulation of amyloid beta in the brain (12, 34, 35, 13, 36). Epileptic patients exhibit elevated tau and amyloid beta levels, possibly linked to disturbances in the excitatory/inhibitory balance (24, 25, 21, 37, 38). Schizophrenia, though not directly linked to amyloid beta pathology, shares a reduction in white matter tract integrity with AD, and patients face higher exposure to risk factors for cognitive decline (39, 40). Attention-Deficit and Hyperactivity Disorder (ADHD) has been associated with increased AD risk in epidemiological and genetic studies (41, 42), yet the underlying mechanisms remain unclear and require further investigation.

The retrospective cohort study reported herein investigated the association between CNS-active drugs, including antidepressants, sedatives, anticonvulsants, antipsychotics, and stimulants, and the incidence of AD and other NDDs including non-AD dementia, PD, MS, and ALS in patients 60 years of age or older. For this analysis, the risk of developing AD or other NDDs starting one year after exposure to CNS-active drugs was assessed using the Mariner US-based population insurance claims dataset. Pharmacologic interventions prescribed to treat neuropsychiatric disorders including antidepressants, sedatives, anticonvulsants, antipsychotics, and stimulants are categorized as CNS-active-drugs. These therapies modulate neurotransmitter signaling through multiple mechanisms that can impact neurodegenerative processes and potentially modulate the risk of developing AD and other NDDs (43–51). Secondary analyses to evaluate the impact of age and duration of treatment were conducted. Moreover, we identified and characterized patients who remained free of AD after drug exposure (responders) and those who developed AD (non-responders).

AD is a progressive disease characterized by impaired cognitive function that ultimately results in loss of autonomy and independent living (52–54). Currently, more than 6 million Americans are affected by AD which, along with other dementias, cost the nation $321 billion dollars in 2022 (54). Presently, select anti-amyloid therapies can slow progression of the disease at early stages (55, 56) but a cure for AD remains elusive. The preclinical phase of AD can begin two decades prior to diagnosis and represents a pivotal window for implementing preventative strategies that target risk factors.

## 2 Research design and methods

### 2.1 Data source

The Mariner dataset used for this analysis contains insurance claims data within the United States, with a population primarily residing in the Southeastern region. The Mariner dataset contains patient demographic characteristics, prescription records, patient diagnosis, and procedure information organized under *Current Procedural Terminology*, *International Classification of Diseases, Ninth Revision* (ICD‐9), and *International Statistical Classification of Diseases and Related Health Problems, Tenth Revision* (ICD‐10) codes (**Supplementary Tables S1**, **S2**), following the International Classification of Diseases. In September 2022, the Mariner database included 151 million patients with claims that went from 2010 to April 2021.

PearlDiver is a research software that facilitates interaction with individual commercial, state-based Medicaid, Medicare stand-alone prescription drug plan, group Medicare Advantage, and individual Medicare Advantage data sets (57).

This report follows the Strengthening the Reporting of Observational Studies in Epidemiology (STROBE) reporting guideline. This study was approved by the University of Arizona Institutional Review Board. Requirements for informed consent were waived as the data were deidentified.

### 2.2 Study design and variables

**Figure 1:**
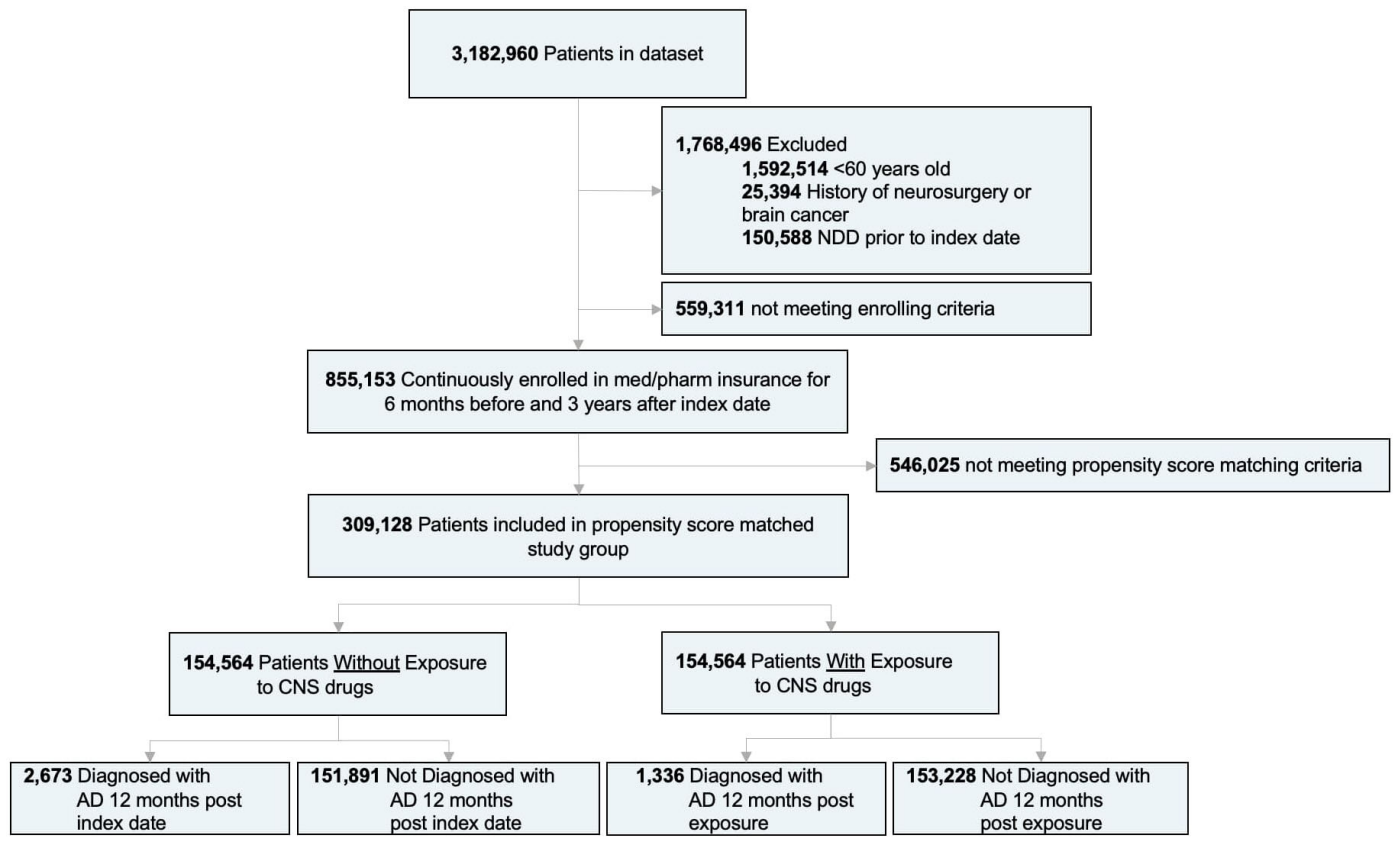
Study design and patient stratification. (AD), Alzheimer’s disease; (NDDs), neurodegenerative diseases.

A subset of patients with non-melanoma skin cancer was selected for medical informatic analysis from the Mariner database as this medical condition is not associated with neurodegenerative disease and treatment for the condition does not require chemotherapy or surgical anesthesia for treatment which can impact risk of Alzheimer’s and occurs with sufficient frequency in the general population to enable robust analyses. Patients younger than 60 years of age, with a history of neurosurgery or brain cancer, and with a history of NDD (including AD, dementia, MS, PD, and ALS) prior to the index date were excluded from this analysis. Participants were required to be continuously enrolled in the medical and pharmacy insurance database for a minimum of 6 months before and 3 years after the index date (**Figure 1**). The index date is described as the first drug prescription record for the treatment group, and a period of 6 months minimum after the first patient claim record for the control group. The treatment group was defined as patients with a medication charge for at least one CNS-active drug, while the control group included patients without any CNS-active drug medication charge throughout the duration of the study. The primary outcome of the study was defined as the incidence of AD, based on ICD-9 and ICD-10 codes, at least 1 year after the index date. The 1-year period is to remove potential unknown effects on NDDs prior to index date. Additionally, incidence of other NDDs including non-AD dementia, MS, PD and ALS was surveyed as exploratory endpoints. Medications considered in the study include FDA-approved antidepressants, sedatives, anticonvulsants, antipsychotics, and stimulants (**Supplementary Table S3**). These drugs were identified by drug codes and then grouped under categories according to their mechanism of action (**Supplementary Table S1**). Age in the treatment group was defined by the age of first CNS-active drug exposure. Following the analysis in (58) and (59) an assessment of comorbidities known to be associated with AD outcomes was conducted. The impact of CNS-active drug duration on the risk of AD was evaluated as described in (60), where analyses were conducted for durations of <1year, 1 to 3 years, 3 to 6 years, and >6 years. Cumulative hazard ratios were built using the propensity score matched population in the Bellwether-PearlDiver interface. The average follow-up time was 6.6 [2.4] years. The number of patients in each drug class and subclass, and the median adherence rate for each therapeutic are reported in **Supplementary Table S3**. A responder analysis was conducted including selected comorbidities known to be associated with AD and other age-related NDDs (61–64). Common co-therapies that have been previously identified as potential AD risk modifiers were also assessed (58–60, 65–68). The drug groups included were antidiabetics, antihypertensives, anti-inflammatories, Selective Estrogen Receptor Modulators (SERMs), Androgen Deprivation Treatments (ADTs), statins and Menopausal Hormonal Treatment (MHT) (**Supplementary Table S1**).

### 2.3 Statistical analysis

Statistical analyses were conducted between May 1st and July 28th, 2022. Patient demographic statistics and incidence statistics were analyzed using unpaired and paired two-tailed t-tests or χ^2^ tests, as appropriate, to test the significance of the differences between continuous and categorical variables. In all analyses, a two-sided p < 0.05 was considered statistically significant.

To balance demographic and comorbidity characteristics between the treatment and control groups, propensity score matching was conducted as described in (58, 59). A logistic regression was generated prior to propensity score matching to estimate the probability for each patient to receive CNS-active drug therapy given confounding variables like age, sex, region, Charlson Comorbidity Index (CCI) score, and comorbidities of interest. The confounding variables that showed statistically significant differences were used to propensity score match the treatment and control groups; these comprised all the demographic variables as well as comorbidities including Asthma, COPD, Cardiac Arrythmia, Cerebrovascular Disease, Chronic Artery Disease, Diabetes, Drug Abuse, Hypertension, Hypothyroidism, Liver Disease, Obesity, Osteoarthritis, Rheumatoid Arthritis, Tobacco Use, Acute Kidney Injury, and Capsulitis.

## 3 Results

Of 3,182,960 patients, a subset of 855,153 met the inclusion and enrollment criteria (**Figure 1**). After adjusting based on age, sex, region, and selected comorbidities, a population of 309,128 patients was equally divided into treatment (mean age [SD]: 66.1 [4.0] years) and control groups (66.1 [4.0] years) (N:154,564). There were no significant differences (p>0.05) on age, sex, or region between adjusted treatment and control groups (**Table 1**). Although the CCI was not significantly different, patients on CNS-active drugs had significantly greater incidence of comorbidities (p < 0.0001) (**Table 1**).

**Table 1:** Baseline characteristics for unadjusted and propensity score-matched patients with or without exposure to CNS active drugs.

	UNADJUSTED COHORT					PROPENSITY SCORE MATCHED COHORT				
	Without Exposure to CNS drugs		With Exposure to CNS drugs			Without Exposure to CNS drugs		With Exposure to CNS drugs		
	n	%	n	%		n	%	n	%	
Number of Patients	252,972		602,181		p value	154,564		154,464		p value
Age										
*60 to 64*	64,761	*25.60%*	287,930	*47.81%*	*0.166*	51,147	*33.11%*	51,147	*33.11%*	*>0.999*
*65 to 69*	57,166	*22.60%*	108,841	*18.07%*		34,685	*22.46%*	34,685	*22.46%*	
*70 to 74*	83,434	*32.98%*	158,592	*26.34%*		51,071	*33.06%*	51,071	*33.06%*	
*75 to 79*	47,611	*18.82%*	46,818	*7.77%*		17,661	*11.43%*	17,661	*11.43%*	
Sex										
*Female*	112,942	*44.65%*	351,186	*58.32%*	*0.222*	75,862	*49.08%*	75,862	*49.11%*	*>0.999*
*Male*	140,030	*55.35%*	250,995	*41.68%*		78,702	*50.92%*	78,702	*50.95%*	
Region										
*Midwest*	56,975	*22.52%*	126,147	*20.95%*	*0.058*	32,880	*21.27%*	32,880	*21.29%*	*>0.999*
*Northeast*	64,594	*25.53%*	131,900	*21.90%*		36,480	*23.60%*	36,480	*23.62%*	
*South*	94,853	*37.50%*	258,840	*42.98%*		64,552	*41.76%*	64,552	*41.79%*	
*West*	36,279	*14.34%*	84,748	*14.07%*		20,624	*13.34%*	20,624	*13.35%*	
*Unknown*	271	*0.11%*	546	*0.09%*		28	*0.02%*	28	*0.02%*	
Comorbidities										
*Cardiovascular Disease*	5,366	*2.12%*	48,060	*7.98%*	*<.0001*	2,255	*1.46%*	8,048	*5.21%*	*<.0001*
*Chronic Kidney Disease*	5,230	*2.07%*	50,239	*8.34%*	*<.0001*	1,948	*1.26%*	8,132	*5.26%*	*<.0001*
*COPD*	3,171	*1.25%*	50,002	*8.30%*	*<.0001*	1,170	*0.76%*	5,812	*3.76%*	*<.0001*
*Diabetes*	7,385	*2.92%*	54,558	*9.06%*	*<.0001*	3,386	*2.19%*	8,690	*5.63%*	*<.0001*
*Hypercholesterolemia*	11,954	*4.73%*	80,438	*13.36%*	*<.0001*	6,983	*4.52%*	17,127	*11.09%*	*<.0001*
*Hypertension*	19,982	*7.90%*	118,033	*19.60%*	*<.0001*	12,632	*8.17%*	29,609	*19.17%*	*<.0001*
*Obesity*	6,835	*2.70%*	71,387	*11.85%*	*<.0001*	6,113	*3.95%*	10,124	*6.55%*	*<.0001*
*Stroke*	4,738	*1.87%*	39,117	*6.50%*	*<.0001*	2,106	*1.36%*	6,781	*4.39%*	*<.0001*
*Tobacco Use*	4,914	*1.94%*	39,442	*6.55%*	*<.0001*	2,325	*1.50%*	5,612	*3.63%*	*<.0001*
CCI										
*0-4*	238,082	*94.11%*	565,322	*93.88%*	*0.385*	152,868	*98.97%*	152,867	*98.97%*	*>0.999*
*5-10ASDASD*	13,864	*5.48%*	34,037	*5.65%*		1,685	*1.09%*	1,686	*1.09%*	
*11+*	1,013	*0.40%*	2,804	*0.47%*		11	*0.01%*	11	*0.01%*	

(AD), Alzheimer’s disease; (CCI), Charlson Comorbidity Index; chronic (COPD), obstructive pulmonary disease.

The majority of patients in the treated group were exposed to either sedatives (38.86%) or antidepressants (33.28%), where the most prevalent drugs were Selective Serotonin Reuptake Inhibitors (SSRIs) (19.56%) and benzodiazepines (BDZPs) (26.73%), respectively (**Supplementary Table S3**). The median adherence for all drug subclasses was greater than 60% except for Z-drugs and BDZPs, which had a 43.34% and 15.33% median adherence, respectively (**Supplementary Table S3**).

Overall, exposure to CNS-active drugs in the propensity score matched population was associated with a significant decrease in the incidence of AD (0.86% vs 1.73%, RR: 0.50; 95% CI: 0.47-0.53; p < 0.0001) and other NDDs (3.13% vs 5.76%, RR: 0.54; 95% CI: 0.53-0.56; p < 0.0001) compared to untreated patients (**Supplementary Figure S1**, **Supplementary Table S4**). The number of patients required to treat to reduce the risk of AD was 115 and for all NDDs combined was 38 (**Supplementary Figure S1**, **Supplementary Table S4**). Sex differences analysis revealed that women treated with CNS-active drugs exhibited a significantly greater NDD risk reduction (RR: 0.50; 95% CI: 0.48-0.53; p < 0.0001) compared to men (RR: 0.59; 95% CI: 0.56-0.61; p < 0.0001), which was driven by non-AD dementia (**Supplementary Figure S2**).

**Figure 2:**
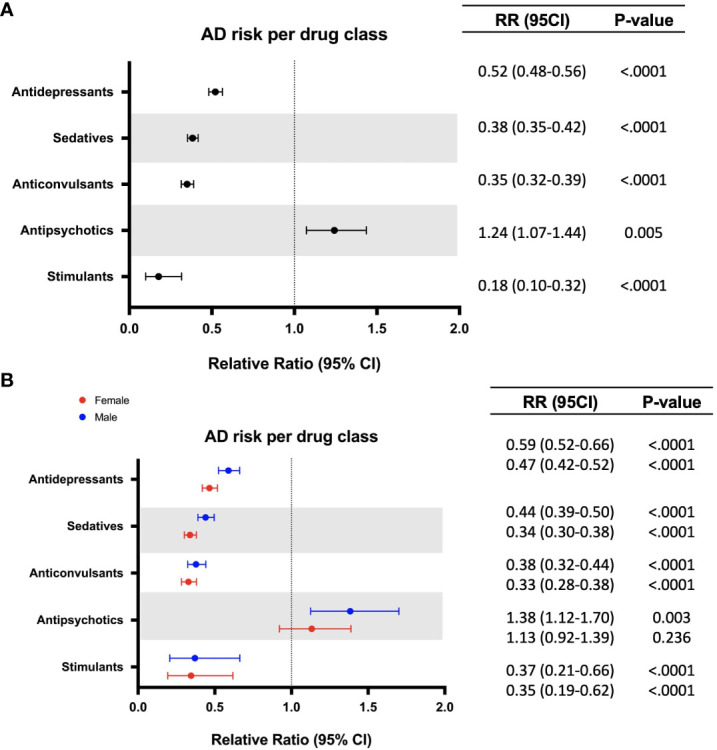
Relative risk of developing Alzheimer’s disease (AD) in patients with exposure to different classes of CNS drugs **(A)**. Sex differences in relative risk of developing Alzheimer’s disease (AD) in patients with exposure to different classes of CNS drugs **(B)** (females appear in red, males appear in blue). CI, confidence interval; RR, relative risk.

To evaluate the association between each CNS-active drug class and AD risk, the treated group was subdivided into five drug classes including antidepressants, sedatives, anticonvulsants, antipsychotics, and stimulants. Stimulants were associated with the greatest risk reduction (0.31% vs 1.73%, RR: 0.18; 95% CI: 0.10-0.32; p < 0.0001) followed by anticonvulsants (0.60% vs 1.73%, RR: 0.35; 95% CI: 0.32-0.39; p < 0.0001), sedatives (0.66% vs 1.73%, RR: 0.38; 95% CI: 0.35-0.42; p < 0.0001), and antidepressants (0.90% vs 1.73%, RR: 0.52; 95% CI: 0.48-0.56; p < 0.0001) (**Table 2**, **Figure 2A**). Antipsychotics were associated with increased AD risk compared to the non-exposure group (2.15% vs 1.73%, RR: 1.24; 95% CI: 1.07-1.44; p < 0.005) (**Table 2**, **Figure 2A**). Analysis of sex differences indicated that sedatives were associated with significantly greater AD risk reduction in women (RR: 0.34; 95% CI: 0.30-0.38; p < 0.0001) compared to men (RR: 0.44; 95% CI: 0.39-0.50; p < 0.0001), whereas antipsychotic association with increased risk of AD was significant only in males (RR: 1.38; 95% CI: 1.12-1.70; p = 0.003) (**Figure 2B**).

**Table 2:** Relative risk of AD development in propensity score matched patients after exposure to different classes of CNS-active drugs.

	Patients with AD diagnosis	%	Relative Risk	95%CI	*p-value*	NNT
No exposure to CNS active drug	2,673	*1.73%*	–	–	–	–
Antidepressants	800	*0.90%*	0.52	0.48-0.56	*<.0001*	121
Sedatives	*687*	*0.66%*	0.38	0.35-0.42	*<.0001*	94
Anticonvulsants	374	*0.60%*	0.35	0.32-0.39	*<.0001*	89
Antipsychotics	*190*	*2.15%*	1.24	1.07-1.44	*0.005*	239
Stimulants	11	*0.31%*	0.18	0.10-0.32	*<.0001*	70

(AD), Alzheimer’s disease; (CI), confidence interval; (RR), relative risk; (NNT), number needed to treat.

**Figure 3:**
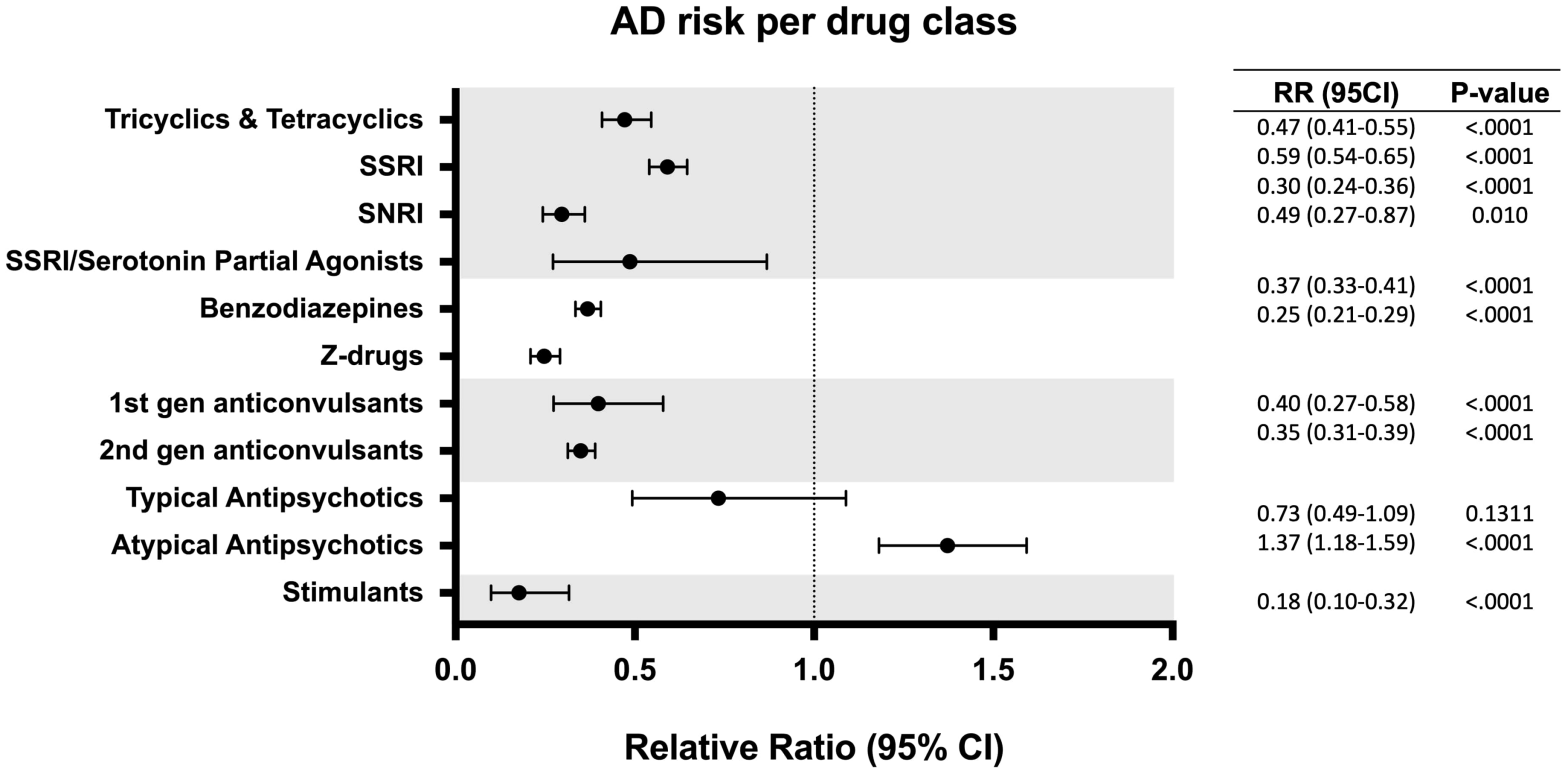
Relative risk of developing Alzheimer’s disease (AD) in patients with exposure to different subclasses of CNS drugs. CI, confidence interval; RR, relative risk.

To determine the impact of specific drugs within a class on AD risk, each drug category was further divided into subclasses. For antidepressant medications, SSRIs were associated with the lowest risk reduction (1.02% vs 1.73%, RR: 0.59; 95% CI: 0.54-0.65; p < 0.0001), followed by tricyclics and tetracyclics (0.82% vs 1.73%, RR: 0.47; 95% CI: 0.41-0.55; p < 0.0001), SSRI/Serotonin Partial Agonists (0.84% vs 1.73%, RR:0.49; 95% CI: 0.27-0.87; p = 0.010) and Serotonin and Norepinephrine Reuptake Inhibitors (SNRIs) (0.51% vs 1.73%, RR: 0.30; 95% CI: 0.24-0.36; p < 0.0001). Within the sedatives category, Z-drugs (0.43% vs 1.73%, RR: 0.25; 95% CI: 0.21-0.29; p < 0.0001) were associated with a significantly greater risk reduction than BDZPs (0.64% vs 1.73%, RR: 0.37; 95% CI: 0.33-0.41; p < 0.0001), which corresponded to the greatest risk reduction among all the drug types assessed. First generation anticonvulsants (0.69% vs 1.73%, RR: 0.40; 95% CI: 0.27-0.58; p < 0.0001) and second generation anticonvulsants (0.60% vs 1.73%, RR: 0.35, 95% CI: 0.31-0.39; p < 0.0001) exhibited a similar risk reduction profile, with the latter inducing slightly greater AD risk reduction as well as reduced variance. Notably, atypical antipsychotics were associated with an increased risk of developing AD (2.37% vs 1.73%, RR:1.37; 95% CI: 1.18-1.59; p < 0.0001) while typical antipsychotics showed no significant change in the association of developing AD (1.27% vs 1.73%, RR: 0.73; 95% CI: 0.49-1.09; p = 0.13) (**Figure 3**).

**Figure 4:**
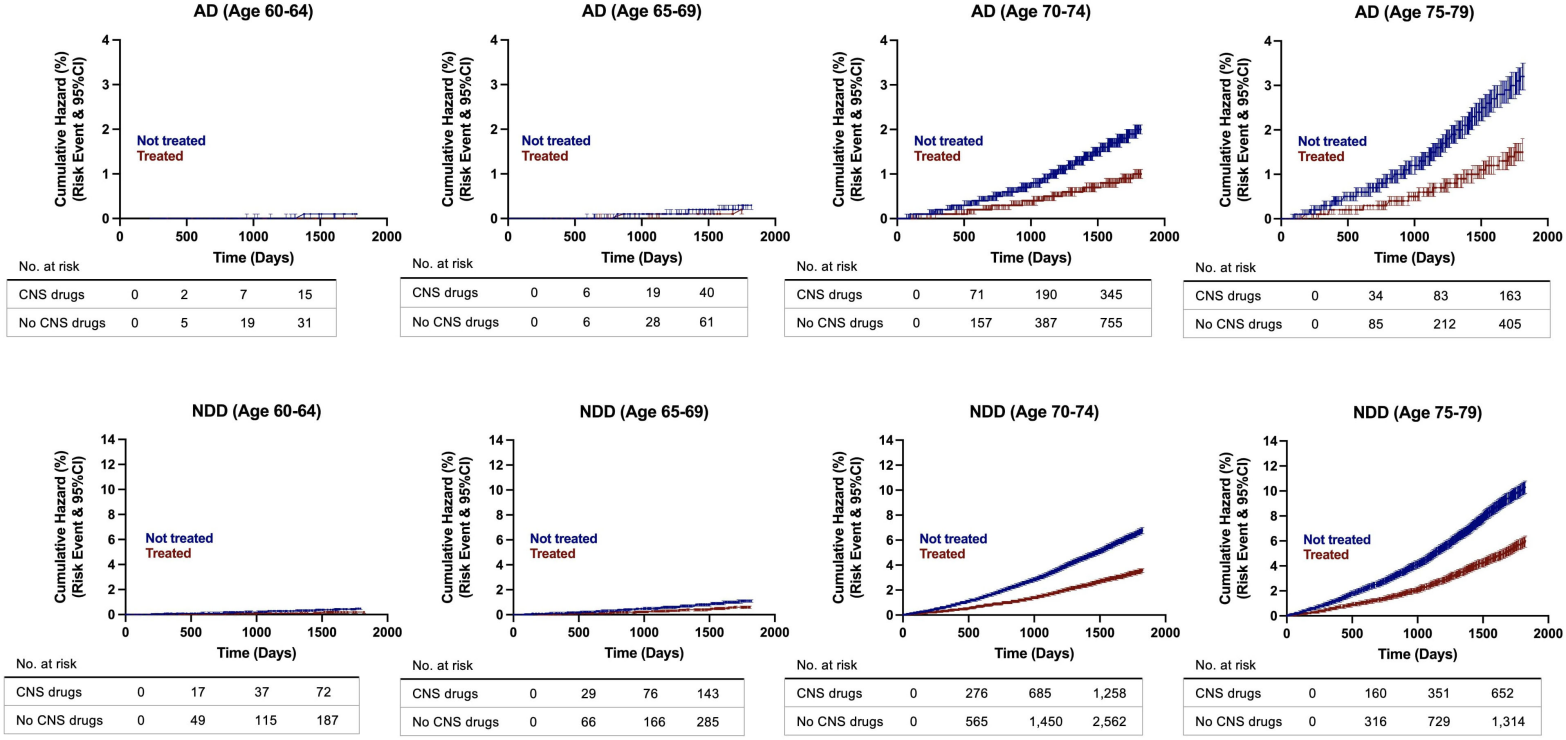
Hazard ratio curves for risk of developing Alzheimer’s disease (AD) and neurodegenerative diseases (NDDs) combined in propensity score matched patients. AD, Alzheimer’s disease; NDD, neurodegenerative disease.

**Figure 5:**
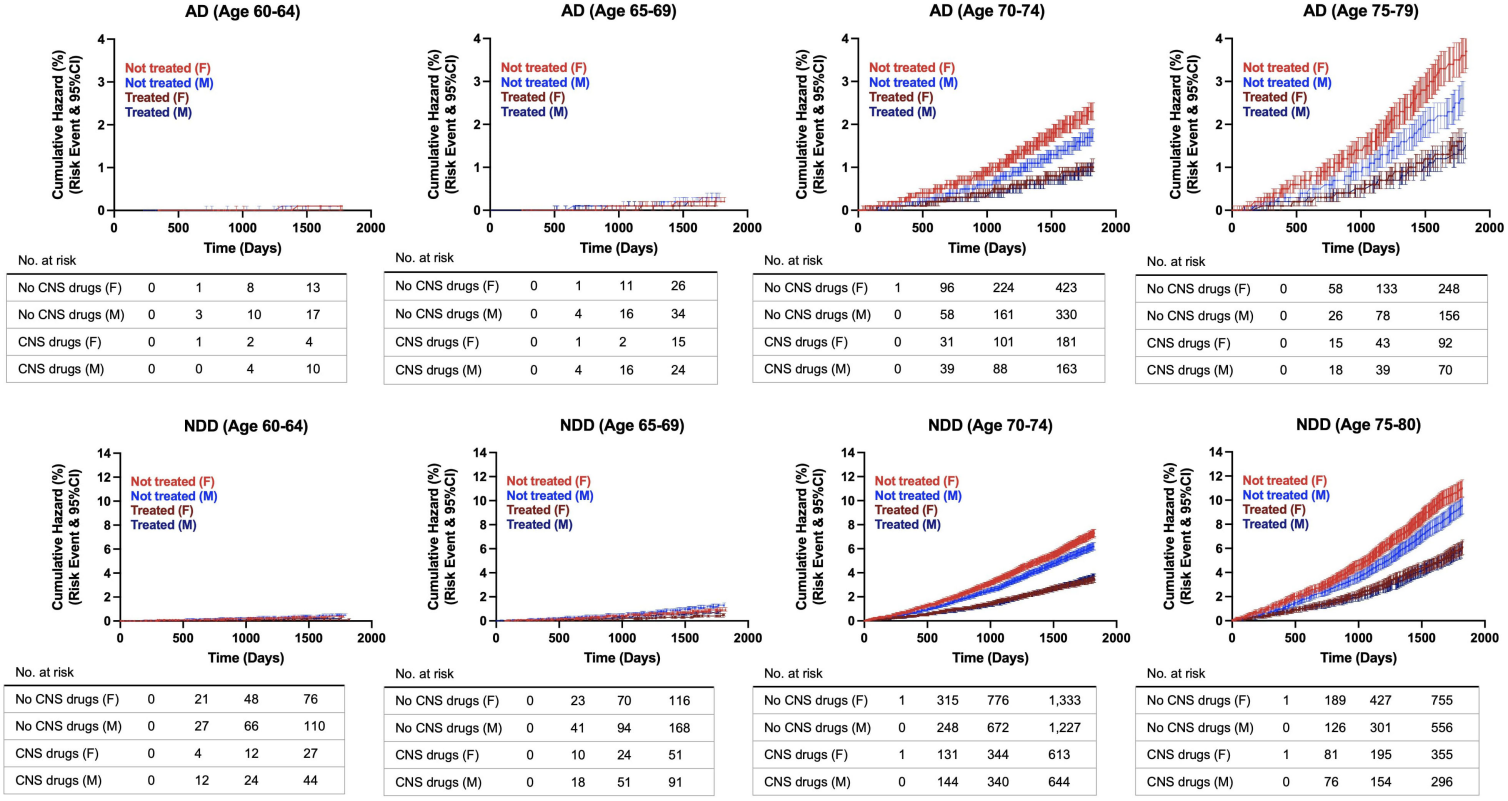
Hazard ratio curves for risk of developing Alzheimer’s disease (AD) and neurodegenerative diseases (NDDs) combined in propensity score matched patients, females versus males. AD, Alzheimer’s disease; NDD, neurodegenerative disease.

Cumulative hazard ratios with 95% CI were generated from the propensity score matched population to evaluate the rate of disease conversion for AD and all NDDs combined. In patients aged 60 to 65 years old, there were no differences between patients with exposure to CNS-active drugs and the control group. However, as age increased, divergence between both groups was greater, with the non-exposure group showing increased risk of developing NDDs and AD compared to the treatment group. The greatest divergence occurred in the oldest group, in patients aged 75 to 79 years old (**Figure 4**). Interestingly, sex differences occurred in patients aged 70 years and older. Females with no exposure to CNS-active drugs had higher risk of developing NDDs and AD compared to males. In contrast, there were no differences in the rate of disease conversion between both sexes in the treated group (**Figure 5**).

The impact of duration of therapy on risk of developing AD and all NDDs combined was determined for patients with exposure to CNS-active drugs in the propensity score matched population (**Table 3**). Within the exposure group, the majority of patients received treatment for 6 years or longer (44.21%). In patients receiving CNS-active drugs for 3 years or more, CNS-active drugs were associated with a significant reduction in AD and NDD risk. Patients receiving CNS-active drugs for over 6 years exhibited the highest risk reduction in AD (RR: 0.22; 95% CI: 0.20 – 0.25; p <0.0001) and NDD (RR: 0.28; 95% CI: 0.27 - 0.30; p <0.0001). In contrast, when treatment duration was less than 3 years, no benefit on AD or NDD risk reduction was detected. A significant increase in AD risk occurred in patients exposed to CNS-active drugs for less than 1 year (RR: 1.23; 95% CI: 1.04-1.47; p = 0.02) as well as an increased risk for NDDs in patients receiving treatment from 1 to 3 years (RR: 1.09; 95% CI: 1.03-1.16; p = 0.0025) (**Table 3**).

**Table 3:** Impact of CNS drug therapy duration on risk of Alzheimer’s disease (AD) and neurodegenerative diseases (NDDs) combined.

	AD			NDDs		
**Duration**	No. (%)	RR (95% CI)	P-value	No. (%)	RR (95% CI)	P-value
1y or less (N = 6,097)	130 (2.13%)	1.23 (1.04-1.47)	0.02	350 (5.74%)	1.00 (0.90-1.11)	0.98
1-3y (N = 19,614)	357 (1.82%)	1.05 (0.94-1.17)	0.35	1,236 (6.30%)	1.09 (1.03-1.16)	0.0025
3-6y (N = 58,143)	582 (1.00%)	0.58 (0.53-0.63)	<.0001	2,125 (3.65%)	0.63 (0.61-0.66)	<.0001
6y and longer (N = 68,338)	265 (0.39%)	0.22 (0.20-0.25)	<.0001	1,113 (1.63%)	0.28 (0.27-0.30)	<.0001

AD, Alzheimer’s disease; CI, confidence interval; NDD, neurodegenerative disease; RR, relative risk.

A responder analysis was conducted to characterize patients who remained free of AD after CNS-active drug exposure (responders) versus patients who developed AD (non-responders) (**Table 4**). Non-responders were older and predominantly female compared to patients who did not develop AD. Non-responders had a significantly higher prevalence of cardiovascular disease (9.4% vs 5.2%, p <.0001), hypertension (26.6% vs 19.1%, p < 0.0001), stroke (9.4% vs 4.4%, p < 0.0001), chronic kidney disease (10.8% vs 5.3%, p < 0.0001), and diabetes (7.3% vs 5.6%, p = 0.009). In contrast, responders were significantly more obese (6.6% vs 2.9%, p < 0.0001). Further, responders were prescribed significantly more MHT (3.5% vs 2.4%, p = 0.025) and anti-inflammatories (30.3% vs 26.1%, p = 0.0006) and less antihypertensive medications (21.2% vs 23.6%, p = 0.037) compared to non-responders (**Table 4**).

**Table 4:** Demographic characteristics, comorbidities and cotreatments in patients who develop Alzheimer’s disease (AD) (non-responders) and those who remain free of AD (responders) after treatment with CNS drugs compared to the untreated population.

	Untreated					Treated				
	Develop AD after 12 m		Do not develop AD after 12 m			Develop AD after 12 m		Do not develop AD after 12 m		
	n	%	n	%		n	%	n	%	
Number of Patients	2,673		151,891		p value	1,336		153,228		p value
Age										
*60 to 64*	78	*2.92%*	51,069	*33.62%*	*0.017*	38	*2.84%*	51,109	*33.35%*	*0.017*
*65 to 69*	166	*6.21%*	34,519	*22.73%*		107	*8.01%*	34,578	*22.57%*	
*70 to 74*	1,838	*68.76%*	49,233	*32.41%*		912	*68.26%*	50,159	*32.73%*	
*75 to 79*	591	*22.11%*	17,070	*11.24%*		279	*20.88%*	17,382	*11.34%*	
Sex										
*Female*	1,495	*55.93%*	74,367	*48.96%*	*0.015*	687	*51.42%*	75,175	*49.06%*	*0.012*
*Male*	1,178	*44.07%*	77,524	*51.04%*		649	*48.58%*	78,053	*50.94%*	
Region										
*Midwest*	627	*23.46%*	32,253	*21.23%*	*0.025*	287	*21.48%*	32,593	*21.27%*	*0.043*
*Northeast*	559	*20.91%*	35,921	*23.65%*		303	*22.68%*	36,177	*23.61%*	
*South*	1159	*43.36%*	63,393	*41.74%*		575	*43.04%*	63,977	*41.75%*	
*West*	328	*12.27%*	20,296	*13.36%*		170	*12.72%*	20,454	*13.35%*	
*Unknown*	-	*0.00%*	28	*0.02%*		11	*0.82%*	27	*0.02%*	
Comorbidities										
*Cardiovascular Disease*	206	*7.71%*	2,193	*1.44%*	*<.0001*	126	*9.43%*	7,957	*5.19%*	*<.0001*
*Chronic Kidney Disease*	224	*8.38%*	1,899	*1.25%*	*<.0001*	144	*10.78%*	8,055	*5.26%*	*<.0001*
*COPD*	120	*4.49%*	1,151	*0.76%*	*<.0001*	57	*4.27%*	5,781	*3.77%*	*0.3481*
*Diabetes*	119	*4.45%*	3,318	*2.18%*	*<.0001*	98	*7.34%*	8,612	*5.62%*	*0.0087*
*Hypercholesterolemia*	229	*8.57%*	6,854	*4.51%*	*<.0001*	150	*11.23%*	17,007	*11.10%*	*0.8612*
*Hypertension*	434	*16.24%*	12,363	*8.14%*	*<.0001*	356	*26.65%*	29,332	*19.14%*	*<.0001*
*Obesity*	64	*2.39%*	3,191	*2.10%*	*0.31*	39	*2.92%*	10,094	*6.59%*	*<.0001*
*Stroke*	182	*6.81%*	2,043	*1.35%*	*<.0001*	126	*9.43%*	6,682	*4.36%*	*<.0001*
*Tobacco Use*	64	*2.39%*	2,274	*1.50%*	*0.0004*	49	*3.67%*	5,567	*3.63%*	*0.9414*
Co-treatments										
*Antidiabetics*	38	*1.42%*	1,726	*1.14%*	*0.168*	84	*6.29%*	9,167	*5.98%*	*0.643*
*Antihypertensives*	186	*6.96%*	7,004	*4.61%*	*<.0001*	315	*23.58%*	32,506	*21.21%*	*0.037*
*Anti-inflammatories*	235	*8.79%*	11,068	*7.29%*	*0.004*	348	*26.05%*	46,471	*30.33%*	*0.0006*
*SERMS*	17	*0.64%*	410	*0.27%*	*0.002*	19	*1.42%*	2,640	*1.72%*	*0.451*
*ADT*	55	*2.06%*	2,718	*1.79%*	*0.303*	84	*6.29%*	11,452	*7.47%*	*0.105*
*Statins*	146	*5.46%*	6,518	*4.29%*	*0.004*	285	*21.33%*	32,083	*20.94%*	*0.736*
*MHT*	13	*0.49%*	939	*0.62%*	*0.455*	32	*2.40%*	5,419	*3.54%*	*0.025*
CCI										
*0-4*	2,630	*98.39%*	150,238	*98.91%*	*0.416*	1,310	*98.05%*	151,557	*98.91%*	*0.416*
*5-10*	22	*0.82%*	1,643	*1.08%*		15	*1.12%*	1,660	*1.08%*	
*11+*	11	*0.41%*	11	*0.01%*		11	*0.82%*	11	*0.01%*	

(AD), Alzheimer’s disease; (ADT), androgen deprivation therapy; CCI, Charlson comorbidity index; (COPD), chronic obstructive pulmonary disease; (MHT), menopausal hormonal treatment; (SERMS), Selective estrogen receptor modulators.

**Figure 6:**
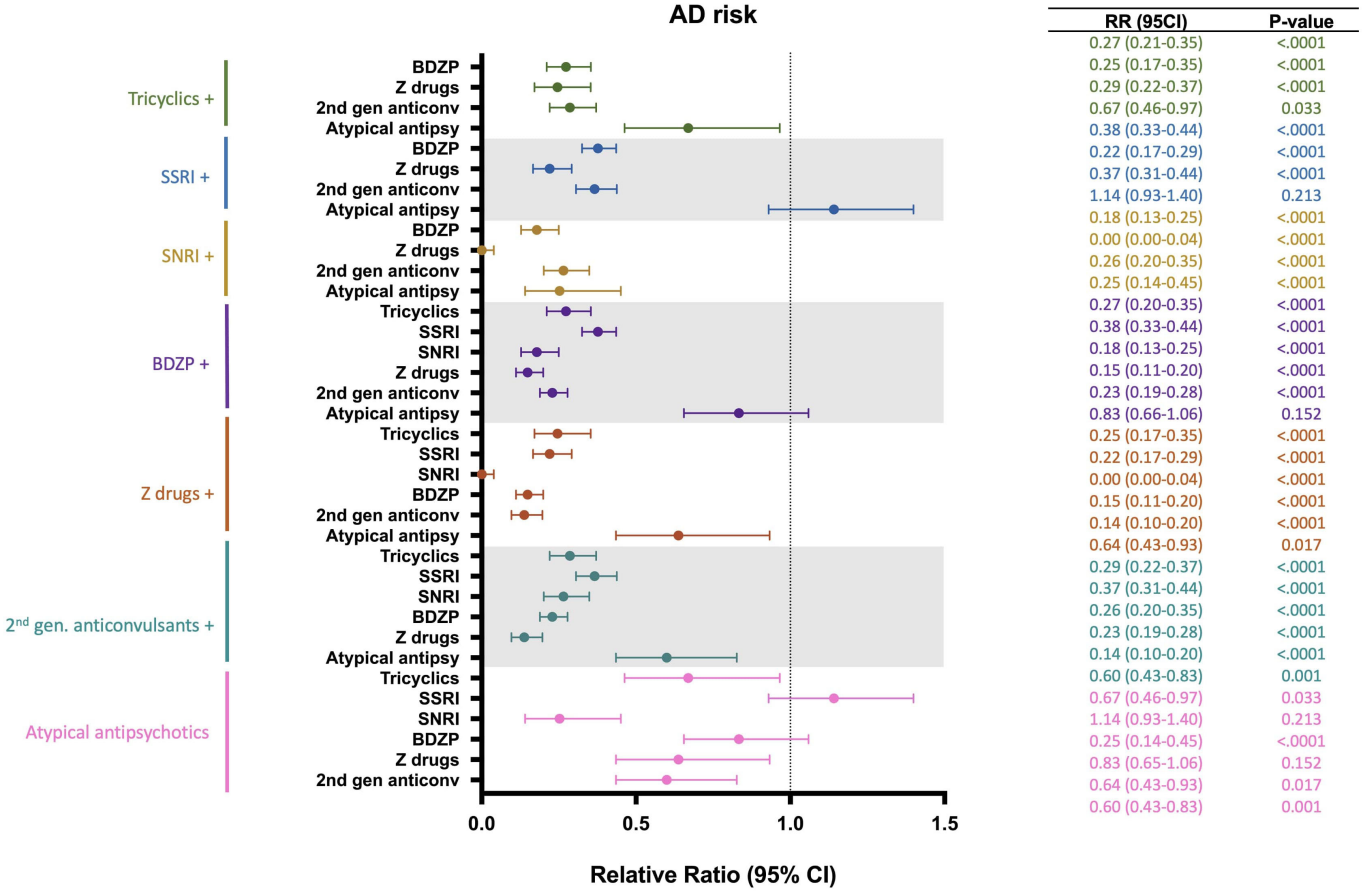
Relative risk of developing Alzheimer’s disease (AD) in patients receiving different combinations of CNS-active drugs. BDZP, benzodiazepines; CI, confidence interval; SNRI, serotonin and norepinephrine reuptake inhibitors; SSRI, selective serotonin reuptake inhibitors.

A risk analysis was conducted to estimate the impact of multiple CNS-active drug combinations on the risk of developing AD (**Figure 6**). Drug groups with insufficient patient number were excluded from this analysis. Outcomes of this analysis indicated that addition of a second CNS-active drug was associated with greater reduction in AD risk compared to monotherapy. Specifically, for all antidepressants assessed (tricyclics, SSRIs and SNRIs), the combination of a BDZP, a Z-drug or a second-generation anticonvulsant was associated with greater reduction of AD risk compared to exposure to one antidepressant alone. BDZP and second-generation anticonvulsants exerted greater reduction in AD risk in all drug combinations explored, except when combined with SSRIs. Similarly, Z-drugs showed decreased AD risk in all combinations with the exception of tricyclic antidepressants. Interestingly, atypical antipsychotics were associated with increased AD risk, which was mitigated by the addition of a tricyclic, SNRI, Z-drug or second-generation anticonvulsant, with Z-drugs providing the greatest mitigation of AD risk.

Addition of a Z-drug to any CNS-active drug was associated with the greatest reduction of AD risk for every drug class assessed, with the combination of a Z-drug and an SNRI exerting greatest AD risk reduction (**Figure 6**).

## 4 Discussion

This study sought to explore the impact of CNS-active medications in an aging population on the development of AD and other age-related NDDs using health claims records. Outcomes of this retrospective analysis indicated that use of CNS-active drugs was associated with significant risk reduction of AD, non-AD dementia, MS, PD, and ALS. Additional analyses revealed that antidepressant, sedative, anticonvulsant, and stimulant medications were associated with a decreased risk of AD, while antipsychotics, specifically atypical antipsychotics, were associated with increased risk of developing AD. Additionally, sedatives were associated with a greater reduction in AD risk in women compared to men, and antipsychotic treatment was associated with increased risk of AD only in males.

Age and duration of therapy emerged as two main modulators of AD risk associated with CNS-active drugs, where AD risk reduction was apparent after 3 years of drug exposure and reduction of AD risk was greater with increasing age. During early stages of AD pathology, a misdiagnosis of a neuropsychiatric disorder can occur as a consequence of the common symptomatology of both diseases (69, 70). Thus, the increased AD risk observed in patients receiving CNS-active drugs for less than 3 years may be the result of AD pathology already established in this group of patients, but not yet diagnosed. These findings suggest that accounting for treatment duration and examining patients in the appropriate age range are key elements of the association between CNS-active drugs and incidence of NDDs. This could also explain disparities in the literature where studies that considered treatment duration indicated that antidepressant and sedative drugs were associated with reduced incidence of dementia (71–73), while studies that did not account for duration of treatment found an increased risk associated with these drugs (74–78).

Risk analysis evaluating sex differences did not reveal significant differences between males and females on AD risk. However, when NDDs were combined a slight but significant difference emerged where females exhibited greater risk reduction compared to males. Consistent with these findings, results from the cumulative hazard analysis indicated a reduction in rate of disease conversion for both sexes in the treated group, with females exhibiting a greater magnitude of divergence between the treated and untreated groups. Within the treated group, females and males exhibited a comparable trajectory of disease conversion suggesting that CNS-active drugs activate mechanisms that reduce risk of NDD that are independent of biological sex.

Results from these analyses indicated that antidepressants, including tricyclics and tetracyclics, SSRIs, SNRIs and SSRI/Serotonin partial agonists, were associated with a significant risk reduction of developing AD. Mechanistically, antidepressants exert multiple actions in the brain including modifications of synaptic transmission, reduced inflammation, and increased neurogenesis that could ameliorate the pathological changes of depression that could increase the risk of developing AD (43, 45, 79–83). Moreover, antidepressants can impact amyloid plaque development (47, 84). The majority of these beneficial effects are thought to be regulated by increases in serotonin and norepinephrine signaling (85).

Sedatives used for the management of insomnia including Z-drugs and BDZPs (86) were associated with reduced risk of developing AD and was greater in females relative to males. Z-drugs were associated with greater risk reduction compared to BDZPs. Both Z-drugs and BDZPs could protect against AD by improving sleep, thereby reducing inflammation, tau aggregation, and amyloid beta accumulation (12, 50, 87). Notably, Z-drugs bind with greater specificity to the GABA-A chloride channel receptor than BDZPs and promote slow-wave sleep, crucial for Aβ clearance and memory consolidation, which could provide an additional benefit in AD risk reduction (88) compared to BDZPs. Interestingly, sedative drugs, especially BDZPs, are highly lipophilic which could induce longer duration of action in women, who have larger volumes of adipose tissue than men (89). Of note, median adherence for both sedative drug classes assessed, specifically for BDZPs, was lower than 50%, which could impact the results on the association between AD risk and treatment with sedative drugs.

Anticonvulsants were associated with reduced AD risk, despite previous studies reporting contradictory findings on cognition and AD risk (90–93). Data contained herein provide new evidence for potential long-term benefits of anticonvulsants. Neuronal hyperexcitability that occurs in the early stages of AD (94) could potentially be counteracted by anticonvulsants as observed in preclinical studies (46, 48).

Atypical antipsychotics were associated with a significant increase in risk of developing AD. Notably, an association with increased risk of AD was only significant in males. Multiple analyses suggest that certain antipsychotic drugs can impair memory and cognitive function with short-term treatment (44, 95, 96) whereas their long-term impact on AD risk was uncertain (97, 98). Findings reported herein provide evidence for an adverse impact on AD risk. Antipsychotic antagonistic effects on neurotransmitters like acetylcholine and serotonin, crucial for memory and learning, could contribute to AD vulnerability (99, 100). Interestingly, women’s psychotic symptoms respond to lower doses of antipsychotics compared to men (101, 102), which could influence the modulation of AD risk differently in females compared to males.

Interestingly, among all CNS-active drugs stimulants were associated with the greatest AD risk reduction. Although the association between stimulants and AD remains poorly understood, evidence indicates that low-dose methamphetamine may promote neuroprotection via sAPP alpha production (51), neuronal differentiation, and synaptic plasticity (49) that likely involves norepinephrine and dopamine mechanisms.

Responder analyses indicated that non-responders were older and predominantly female compared to patients who did not develop AD which could contribute to the greater incidence of AD in women (54). Additionally, non-responders were characterized by an overall higher incidence of comorbidities than responders, specifically cardiovascular comorbidities, whereas responders had a higher incidence of obesity. Consistent with these findings, previous reports indicate that weight loss at midlife is a risk factor for AD, and that high body mass index in late life could be protective against AD (103–105). Interestingly, responders were also characterized by a greater exposure to anti-inflammatory drugs and MHT which have both been associated with decreased risk of developing AD (60, 65, 106).

## 5 Limitations

Several limitations of this study are due to the Mariner database which does not include values for laboratory tests or APOE genotype information or socioeconomic factors like race, ethnicity, or level of education or lifestyle modifications or psychological therapies which are recommended for several of the neuropsychiatric disorders assessed. Although the groups were propensity score matched, these factors could have an impact on AD development which is not accounted for in the analyses. Further, there was no information regarding efficacy of CNS-active drugs to control the neuropsychiatric disorder or whether therapies were changed during the course of treatment.

The average median adherence for all drugs considered in the study was 71.88%, which is higher than the estimated adherence to chronic medications in the general population (50%) 107. Because all main analyses were conducted with all drugs grouped together, adherence was sufficient to provide reliable results. However, low adherence rate would specifically impact results derived from drug classes with low median adherence. Both sedative drug classes had an adherence rate lower than 50%, with 15.33% for benzodiazepines and 43.34% for Z-drugs. The low adherence rate would underestimate the magnitude of impact of these drugs on NDD risk.

## 6 Conclusion

Outcomes of analyses reported herein indicate that CNS-active drugs targeting neuropsychiatric disorders including depression, insomnia, epilepsy, schizophrenia, and ADHD can significantly modify the risk of developing AD. Antidepressants, sedatives, anticonvulsants, and stimulants, were all associated with significant reduction in the risk of AD, especially following long-term treatment. In contrast, antipsychotics, specifically atypical antipsychotics, were associated with a significant increased risk of developing AD, which could be mitigated when combined with other CNS-active drugs such as SNRIs. Women and men exhibited distinct response profiles to sedative and antipsychotic drugs, where sedatives were associated with a greater reduction in AD risk in women compared to men whereas antipsychotic treatment was associated with increased risk in AD only in males. Notably, non-responders to these therapeutics were characterized by a high incidence of cardiovascular disorders. Collectively, the data indicate that long-term management of neuropsychiatric disorders using appropriate pharmacologic treatment can significantly reduce risk of AD and other NDDs.

## Data availability statement

The datasets presented in this article are not readily available because restrictions apply to the availability of some or all data generated or analyzed during this study to preserve patient confidentiality or because they were used under license. The corresponding author will on request detail the restrictions and any conditions under which access to some data may be provided. Requests to access the datasets should be directed to info@pearldiverinc.com.

## Ethics statement

Ethical approval was not required for the study involving humans in accordance with the local legislation and institutional requirements. Written informed consent to participate in this study was not required from the participants or the participants’ legal guardians/next of kin in accordance with the national legislation and the institutional requirements.

## Author contributions

HC-F: Conceptualization, Formal analysis, Writing – original draft, Writing – review & editing. GT-H: Conceptualization, Supervision, Writing – review & editing. RB: Conceptualization, Funding acquisition, Resources, Supervision, Writing – review & editing.

## References

[1] Poletti M Nuti A Cipriani G Bonuccelli U. Behavioral and psychological symptoms of dementia: factor analysis and relationship with cognitive impairment. Eur Neurol. (2013) 69:76–82. doi: 10.1159/000341956 23154430

[2] Van Der Mussele S Fransen E Struyfs H Luyckx J Marien P Saerens J. Depression in mild cognitive impairment is associated with progression to Alzheimer's disease: a longitudinal study. J Alzheimers Dis. (2014) 42:1239–50. doi: 10.3233/JAD-140405 25024328

[3] Kohler CA Magalhaes TF Oliveira JM Alves GS Knochel C Oertel-Knochel V. Neuropsychiatric disturbances in mild cognitive impairment (MCI): A systematic review of population-based studies. Curr Alzheimer Res. (2016) 13:1066–82. doi: 10.2174/1567205013666160502123129 27137220

[4] Postuma RB Berg D. Prodromal parkinson's disease: the decade past, the decade to come. Mov Disord. (2019) 34:665–75. doi: 10.1002/mds.27670 30919499

[5] Dujardin K Sgambato V. Neuropsychiatric disorders in parkinson's disease: what do we know about the role of dopaminergic and non-dopaminergic systems? Front Neurosci. (2020) 14:25. doi: 10.3389/fnins.2020.00025 32063833 PMC7000525

[6] Makhani N Tremlett H. The multiple sclerosis prodrome. Nat Rev Neurol. (2021) 17:515–21. doi: 10.1038/s41582-021-00519-3 PMC8324569 34155379

[7] Vienažindytė I Cesarskaja J Vaičiulytė D Balnytė R Matijošaitis V. Do prodrome symptoms influence multiple sclerosis disease course and severity? Med Hypotheses. (2022) 165:110888. doi: 10.1016/j.mehy.2022.110888

[8] Roos E Mariosa D Ingre C Lundholm C Wirdefeldt K Roos PM. Depression in amyotrophic lateral sclerosis. Neurology. (2016) 86:2271–7. doi: 10.1212/WNL.0000000000002671 PMC4909561 27164661

[9] Bauer ME Teixeira AL. Inflammation in psychiatric disorders: what comes first? Ann N Y Acad Sci. (2019) 1437:57–67. doi: 10.1111/nyas.13712 29752710

[10] Ochneva A Zorkina Y Abramova O Pavlova O Ushakova V Morozova A. Protein misfolding and aggregation in the brain: common pathogenetic pathways in neurodegenerative and mental disorders. Int J Mol Sci. (2022) 23(22):14498. doi: 10.3390/ijms232214498 PMC9695177 36430976

[11] Brites D Fernandes A. Neuroinflammation and depression: microglia activation, extracellular microvesicles and microRNA dysregulation. Front Cell Neurosci. (2015) 9:476. doi: 10.3389/fncel.2015.00476 26733805 PMC4681811

[12] Irwin MR Olmstead R Carroll JE. Sleep disturbance, sleep duration, and inflammation: A systematic review and meta-analysis of cohort studies and experimental sleep deprivation. Biol Psychiatry. (2016) 80:40–52. doi: 10.1016/j.biopsych.2015.05.014 26140821 PMC4666828

[13] Cordone S Annarumma L Rossini PM De Gennaro L. Sleep and beta-amyloid deposition in alzheimer disease: insights on mechanisms and possible innovative treatments. Front Pharmacol. (2019) 10:695. doi: 10.3389/fphar.2019.00695 31281257 PMC6595048

[14] Osimo EF Pillinger T Rodriguez IM Khandaker GM Pariante CM Howes OD. Inflammatory markers in depression: A meta-analysis of mean differences and variability in 5,166 patients and 5,083 controls. Brain Behav Immun. (2020) 87:901–9. doi: 10.1016/j.bbi.2020.02.010 PMC7327519 32113908

[15] Hyung WSW Kang J Kim J Lee S Youn H Ham BJ. Cerebral amyloid accumulation is associated with distinct structural and functional alterations in the brain of depressed elders with mild cognitive impairment. J Affect Disord. (2021) 281:459–66. doi: 10.1016/j.jad.2020.12.049 33360748

[16] Wang H He Y Sun Z Ren S Liu M Wang G. Microglia in depression: an overview of microglia in the pathogenesis and treatment of depression. J Neuroinflamm. (2022) 19:132. doi: 10.1186/s12974-022-02492-0 PMC9168645 35668399

[17] Boskovic M Vovk T Kores Plesnicar B Grabnar I. Oxidative stress in schizophrenia. Curr Neuropharmacol. (2011) 9:301–12. doi: 10.2174/157015911795596595 PMC3131721 22131939

[18] Gulec M Ozkol H Selvi Y Tuluce Y Aydin A Besiroglu L. Oxidative stress in patients with primary insomnia. Prog Neuropsychopharmacol Biol Psychiatry. (2012) 37:247–51. doi: 10.1016/j.pnpbp.2012.02.011 22401887

[19] Guo C Sun L Chen X Zhang D. Oxidative stress, mitochondrial damage and neurodegenerative diseases. Neural Regener Res. (2013) 8:2003–14. doi: 10.3969/j.issn.1673-5374.2013.21.009 PMC4145906 25206509

[20] Kaufmann T Van Der Meer D Doan NT Schwarz E Lund MJ Agartz I. Common brain disorders are associated with heritable patterns of apparent aging of the brain. Nat Neurosci. (2019) 22:1617–23. doi: 10.1038/s41593-019-0471-7 PMC6823048 31551603

[21] Vico Varela E Etter G Williams S. Excitatory-inhibitory imbalance in Alzheimer's disease and therapeutic significance. Neurobiol Dis. (2019) 127:605–15. doi: 10.1016/j.nbd.2019.04.010 30999010

[22] Gatchel JR Donovan NJ Locascio JJ Schultz AP Becker JA Chhatwal J. Depressive symptoms and tau accumulation in the inferior temporal lobe and entorhinal cortex in cognitively normal older adults: A pilot study. J Alzheimers Dis. (2017) 59:975–85. doi: 10.3233/JAD-170001 PMC5577564 28697559

[23] Li P Hsiao IT Liu CY Chen CH Huang SY Yen TC. Beta-amyloid deposition in patients with major depressive disorder with differing levels of treatment resistance: a pilot study. EJNMMI Res. (2017) 7:24. doi: 10.1186/s13550-017-0273-4 28324341 PMC5360749

[24] Romoli M Sen A Parnetti L Calabresi P Costa C. Amyloid-beta: a potential link between epilepsy and cognitive decline. Nat Rev Neurol. (2021) 17:469–85. doi: 10.1038/s41582-021-00505-9 34117482

[25] Hwang K Vaknalli RN Addo-Osafo K Vicente M Vossel K. Tauopathy and epilepsy comorbidities and underlying mechanisms. Front Aging Neurosci. (2022) 14:903973. doi: 10.3389/fnagi.2022.903973 35923547 PMC9340804

[26] Lv YN Cui Y Zhang B Huang SM. Sleep deficiency promotes Alzheimer's disease development and progression. Front Neurol. (2022) 13:1053942. doi: 10.3389/fneur.2022.1053942 36588906 PMC9795181

[27] Dafsari FS Jessen F. Depression-an underrecognized target for prevention of dementia in Alzheimer's disease. Transl Psychiatry. (2020) 10:160. doi: 10.1038/s41398-020-0839-1 32433512 PMC7239844

[28] Miranda M Morici JF Zanoni MB Bekinschtein P. Brain-derived neurotrophic factor: A key molecule for memory in the healthy and the pathological brain. Front Cell Neurosci. (2019) 13:363. doi: 10.3389/fncel.2019.00363 31440144 PMC6692714

[29] Morgese MG Trabace L. Monoaminergic system modulation in depression and alzheimer's disease: A new standpoint? Front Pharmacol. (2019) 10:483. doi: 10.3389/fphar.2019.00483 31156428 PMC6533589

[30] Martinowich K Lu B. Interaction between BDNF and serotonin: role in mood disorders. Neuropsychopharmacology. (2008) 33:73–83. doi: 10.1038/sj.npp.1301571 17882234

[31] Svob Strac D Pivac NS Muck-Seler D. The serotonergic system and cognitive function. Transl Neurosci. (2016) 7:35–49. doi: 10.1515/tnsci-2016-0007 28123820 PMC5017596

[32] Heneka MT Ramanathan M Jacobs AH Dumitrescu-Ozimek L Bilkei-Gorzo A Debeir T. Locus ceruleus degeneration promotes Alzheimer pathogenesis in amyloid precursor protein 23 transgenic mice. J Neurosci. (2006) 26:1343–54. doi: 10.1523/JNEUROSCI.4236-05.2006 PMC6675491 16452658

[33] Chen Y Chen T Hou R. Locus coeruleus in the pathogenesis of Alzheimer's disease: A systematic review. Alzheimers Dement (N Y). (2022) 8:e12257. doi: 10.1002/trc2.12257 35282658 PMC8900465

[34] Hurtado-Alvarado G Dominguez-Salazar E Pavon L Velazquez-Moctezuma J Gomez-Gonzalez B. Blood-brain barrier disruption induced by chronic sleep loss: low-grade inflammation may be the link. J Immunol Res. (2016) 2016:4576012. doi: 10.1155/2016/4576012 27738642 PMC5050358

[35] Sweeney MD Sagare AP Zlokovic BV. Blood-brain barrier breakdown in Alzheimer disease and other neurodegenerative disorders. Nat Rev Neurol. (2018) 14:133–50. doi: 10.1038/nrneurol.2017.188 PMC5829048 29377008

[36] Giese M Unternahrer E Huttig H Beck J Brand S Calabrese P. BDNF: an indicator of insomnia? Mol Psychiatry. (2014) 19:151–2. doi: 10.1038/mp.2013.10 PMC3903111 23399916

[37] Barker-Haliski M White HS. Glutamatergic mechanisms associated with seizures and epilepsy. Cold Spring Harb Perspect Med. (2015) 5:a022863. doi: 10.1101/cshperspect.a022863 26101204 PMC4526718

[38] Busche MA Konnerth A. Impairments of neural circuit function in Alzheimer's disease. Philos Trans R Soc Lond B Biol Sci. (2016) 371(1700):20150429. doi: 10.1098/rstb.2015.0429 PMC4938029 27377723

[39] Cai L Huang J. Schizophrenia and risk of dementia: a meta-analysis study. Neuropsychiatr Dis Treat. (2018) 14:2047–55. doi: 10.2147/NDT.S172933 PMC6095111 30147318

[40] Kochunov P Zavaliangos-Petropulu A Jahanshad N Thompson PM Ryan MC Chiappelli J. A white matter connection of schizophrenia and alzheimer's disease. Schizophr Bull. (2021) 47:197–206. doi: 10.1093/schbul/sbaa078 32681179 PMC7825012

[41] Zhang L Du Rietz E Kuja-Halkola R Dobrosavljevic M Johnell K Pedersen NL. Attention-deficit/hyperactivity disorder and Alzheimer's disease and any dementia: A multi-generation cohort study in Sweden. Alzheimers Dement. (2022) 18:1155–63. doi: 10.1002/alz.12462 34498801

[42] Leffa DT Ferrari-Souza JP Bellaver B Tissot C Ferreira PCL Brum WS. Genetic risk for attention-deficit/hyperactivity disorder predicts cognitive decline and development of Alzheimer's disease pathophysiology in cognitively unimpaired older adults. Mol Psychiatry. (2023) 28:1248–55. doi: 10.1038/s41380-022-01867-2 36476732

[43] Malberg JE Eisch AJ Nestler EJ Duman RS. Chronic antidepressant treatment increases neurogenesis in adult rat hippocampus. J Neurosci. (2000) 20:9104–10. doi: 10.1523/JNEUROSCI.20-24-09104.2000 PMC6773038 11124987

[44] Minzenberg MJ Poole JH Benton C Vinogradov S. Association of anticholinergic load with impairment of complex attention and memory in schizophrenia. Am J Psychiatry. (2004) 161:116–24. doi: 10.1176/appi.ajp.161.1.116 14702259

[45] Hashioka S Mcgeer P Monji A Kanba S. Anti-inflammatory effects of antidepressants: possibilities for preventives against alzheimers disease. Cent Nervous System Agents Medicinal Chem. (2009) 9:12–9. doi: 10.2174/187152409787601897 20021334

[46] Long Z Zheng M Zhao L Xie P Song C Chu Y. Valproic acid attenuates neuronal loss in the brain of APP/PS1 double transgenic Alzheimer's disease mice model. Curr Alzheimer Res. (2013) 10:261–9. doi: 10.2174/1567205011310030005 23036022

[47] Sheline YI West T Yarasheski K Swarm R Jasielec MS Fisher JR. An antidepressant decreases CSF Abeta production in healthy individuals and in transgenic AD mice. Sci Transl Med. (2014) 6:236re4. doi: 10.1126/scitranslmed.3008169 PMC4269372 24828079

[48] Yao ZG Liang L Liu Y Zhang L Zhu H Huang L. Valproate improves memory deficits in an Alzheimer's disease mouse model: investigation of possible mechanisms of action. Cell Mol Neurobiol. (2014) 34:805–12. doi: 10.1007/s10571-013-0012-y PMC11488907 24939432

[49] Baptista S Lourenco J Milhazes N Borges F Silva AP Bacci A. Long-term treatment with low doses of methamphetamine promotes neuronal differentiation and strengthens long-term potentiation of glutamatergic synapses onto dentate granule neurons. eNeuro. (2016) 3(3). doi: 10.1523/ENEURO.0141-16.2016 PMC4939399 27419216

[50] Holth J Patel T Holtzman DM. Sleep in alzheimer's disease - beyond amyloid. Neurobiol Sleep Circadian Rhythms. (2017) 2:4–14. doi: 10.1016/j.nbscr.2016.08.002 28217760 PMC5312809

[51] Shukla M Maitra S Hernandez JF Govitrapong P Vincent B. Methamphetamine regulates betaAPP processing in human neuroblastoma cells. Neurosci Lett. (2019) 701:20–5. doi: 10.1016/j.neulet.2019.02.023 30771376

[52] Amieva H Le Goff M Millet X Orgogozo JM Peres K Barberger-Gateau P. Prodromal Alzheimer's disease: successive emergence of the clinical symptoms. Ann Neurol. (2008) 64:492–8. doi: 10.1002/ana.21509 19067364

[53] National Institute On Aging, N. Basics of Alzheimer’s Disease and Dementia. NIH, National Institute on Aging: nia.nih.gov (NIH official website) (2021).

[54] Alzheimer’s Association, A. Alzheimer’s disease facts and figures. Alzheimer’s Dement (2022). 10.1002/alz.12638 35289055

[55] Budd Haeberlein S Aisen PS Barkhof F Chalkias S Chen T Cohen S. Two randomized phase 3 studies of aducanumab in early alzheimer's disease. J Prev Alzheimers Dis. (2022) 9:197–210. doi: 10.14283/jpad.2022.30 35542991

[56] Van Dyck CH Swanson CJ Aisen P Bateman RJ Chen C Gee M. Lecanemab in early alzheimer's disease. N Engl J Med. (2023) 388:9–21. doi: 10.1056/NEJMoa2212948 36449413

[57] Pearldiver Technologies, C. S., Co. USA PearlDiver Mariner Patient Claims Database.

[58] Branigan GL Soto M Neumayer L Rodgers K Brinton RD. Association between hormone-modulating breast cancer therapies and incidence of neurodegenerative outcomes for women with breast cancer. JAMA Netw Open. (2020) 3:e201541. doi: 10.1001/jamanetworkopen.2020.1541 32207833 PMC7093781

[59] Torrandell-Haro G Branigan GL Vitali F Geifman N Zissimopoulos JM Brinton RD. Statin therapy and risk of Alzheimer's and age-related neurodegenerative diseases. Alzheimers Dement (N Y). (2020) 6:e12108. doi: 10.1002/trc2.12108 33283039 PMC7687291

[60] Kim YJ Soto M Branigan GL Rodgers K Brinton RD. Association between menopausal hormone therapy and risk of neurodegenerative diseases: Implications for precision hormone therapy. Alzheimers Dement (N Y). (2021) 7:e12174. doi: 10.1002/trc2.12174 34027024 PMC8118114

[61] Fan YC Hsu JL Tung HY Chou CC Bai CH. Increased dementia risk predominantly in diabetes mellitus rather than in hypertension or hyperlipidemia: a population-based cohort study. Alzheimers Res Ther. (2017) 9:7. doi: 10.1186/s13195-017-0236-z 28162091 PMC5292809

[62] Tini G Scagliola R Monacelli F La Malfa G Porto I Brunelli C. Alzheimer's disease and cardiovascular disease: A particular association. Cardiol Res Pract. (2020) 2020:2617970. doi: 10.1155/2020/2617970 32454996 PMC7222603

[63] Wang J Li X Lei S Zhang D Zhang S Zhang H. Risk of dementia or cognitive impairment in COPD patients: A meta-analysis of cohort studies. Front Aging Neurosci. (2022) 14:962562. doi: 10.3389/fnagi.2022.962562 36158542 PMC9500359

[64] Stocker H Beyer L Trares K Perna L Rujescu D Holleczek B. Association of kidney function with development of alzheimer disease and other dementias and dementia-related blood biomarkers. JAMA Netw Open. (2023) 6:e2252387. doi: 10.1001/jamanetworkopen.2022.52387 36692879 PMC10408272

[65] Broe GA Grayson DA Creasey HM Waite LM Casey BJ Bennett HP. Anti-inflammatory drugs protect against Alzheimer disease at low doses. Arch Neurol. (2000) 57:1586–91. doi: 10.1001/archneur.57.11.1586 11074790

[66] Yasar S Xia J Yao W Furberg CD Xue QL Mercado CI. Antihypertensive drugs decrease risk of Alzheimer disease: Ginkgo Evaluation of Memory Study. Neurology. (2013) 81:896–903. doi: 10.1212/WNL.0b013e3182a35228 23911756 PMC3885216

[67] Jayadevappa R Chhatre S Malkowicz SB Parikh RB Guzzo T Wein AJ. Association between androgen deprivation therapy use and diagnosis of dementia in men with prostate cancer. JAMA Netw Open. (2019) 2:e196562. doi: 10.1001/jamanetworkopen.2019.6562 31268539 PMC6613289

[68] Torrandell-Haro G Branigan GL Brinton RD Rodgers KE. Association between specific type 2 diabetes therapies and risk of alzheimer's disease and related dementias in propensity-score matched type 2 diabetic patients. Front Aging Neurosci. (2022) 14:878304. doi: 10.3389/fnagi.2022.878304 35601622 PMC9120543

[69] Lyketsos CG Carrillo MC Ryan JM Khachaturian AS Trzepacz P Amatniek J. Neuropsychiatric symptoms in Alzheimer's disease. Alzheimers Dement. (2011) 7:532–9. doi: 10.1016/j.jalz.2011.05.2410 PMC3299979 21889116

[70] Li XL Hu N Tan MS Yu JT Tan L. Behavioral and psychological symptoms in Alzheimer's disease. BioMed Res Int. (2014) 2014:927804. doi: 10.1155/2014/927804 25133184 PMC4123596

[71] Bartels C Belz M Vogelgsang J Hessmann P Bohlken J Wiltfang J. To be continued? Long-term treatment effects of antidepressant drug classes and individual antidepressants on the risk of developing dementia: A german case-control study. J Clin Psychiatry. (2020) 81(5):19. doi: 10.4088/JCP.19m13205 32857931

[72] Bietry FA Pfeil AM Reich O Schwenkglenks M Meier CR. Benzodiazepine use and risk of developing alzheimer's disease: A case-control study based on swiss claims data. CNS Drugs. (2017) 31:245–51. doi: 10.1007/s40263-016-0404-x 28078633

[73] Guo F Yi L Zhang W Bian ZJ Zhang YB. Association between Z drugs use and risk of cognitive impairment in middle-aged and older patients with chronic insomnia. Front Hum Neurosci. (2021) 15:775144. doi: 10.3389/fnhum.2021.775144 34955792 PMC8696350

[74] Goveas JS Hogan PE Kotchen JM Smoller JW Denburg NL Manson JE. Depressive symptoms, antidepressant use, and future cognitive health in postmenopausal women: the Women's Health Initiative Memory Study. Int Psychogeriatr. (2012) 24:1252–64. doi: 10.1017/S1041610211002778 PMC5800401 22301077

[75] Then CK Chi NF Chung KH Kuo L Liu KH Hu CJ. Risk analysis of use of different classes of antidepressants on subsequent dementia: A nationwide cohort study in Taiwan. PloS One. (2017) 12:e0175187. doi: 10.1371/journal.pone.0175187 28384235 PMC5383251

[76] Tapiainen V Taipale H Tanskanen A Tiihonen J Hartikainen S Tolppanen AM. The risk of Alzheimer's disease associated with benzodiazepines and related drugs: a nested case-control study. Acta Psychiatr Scand. (2018) 138:91–100. doi: 10.1111/acps.12909 29851063

[77] Lee J Jung SJ Choi JW Shin A Lee YJ. Use of sedative-hypnotics and the risk of Alzheimer's dementia: A retrospective cohort study. PloS One. (2018) 13:e0204413. doi: 10.1371/journal.pone.0204413 30248129 PMC6152975

[78] Cheng HT Lin FJ Erickson SR Hong JL Wu CH. The association between the use of zolpidem and the risk of alzheimer's disease among older people. J Am Geriatr Soc. (2017) 65:2488–95. doi: 10.1111/jgs.15018 28884784

[79] Vollmar P Haghikia A Dermietzel R Faustmann PM. Venlafaxine exhibits an anti-inflammatory effect in an inflammatory co-culture model. Int J Neuropsychopharmacol. (2008) 11:111–7. doi: 10.1017/S1461145707007729 17445357

[80] Lee BH Kim YK. The roles of BDNF in the pathophysiology of major depression and in antidepressant treatment. Psychiatry Investig. (2010) 7:231–5. doi: 10.4306/pi.2010.7.4.231 PMC3022308 21253405

[81] Quesseveur G David DJ Gaillard MC Pla P Wu MV Nguyen HT. BDNF overexpression in mouse hippocampal astrocytes promotes local neurogenesis and elicits anxiolytic-like activities. Transl Psychiatry. (2013) 3:e253. doi: 10.1038/tp.2013.30 23632457 PMC3641417

[82] Walker FR. A critical review of the mechanism of action for the selective serotonin reuptake inhibitors: do these drugs possess anti-inflammatory properties and how relevant is this in the treatment of depression? Neuropharmacology. (2013) 67:304–17. doi: 10.1016/j.neuropharm.2012.10.002 23085335

[83] Patricio P Mateus-Pinheiro A Irmler M Alves ND MaChado-Santos AR Morais M. Differential and converging molecular mechanisms of antidepressants' action in the hippocampal dentate gyrus. Neuropsychopharmacology. (2015) 40:338–49. doi: 10.1038/npp.2014.176 PMC4443946 25035085

[84] Cirrito JR Disabato BM Restivo JL Verges DK Goebel WD Sathyan A. Serotonin signaling is associated with lower amyloid-beta levels and plaques in transgenic mice and humans. Proc Natl Acad Sci USA. (2011) 108:14968–73. doi: 10.1073/pnas.1107411108 PMC3169155 21873225

[85] Kim HJ Kim W Kong SY. Antidepressants for neuro-regeneration: from depression to Alzheimer's disease. Arch Pharm Res. (2013) 36:1279–90. doi: 10.1007/s12272-013-0238-8 24129616

[86] Schutte-Rodin S Buysse L Dorsey D Sateia M C. Clinical guideline for the evaluation and management of chronic insomnia in adults. Clin Sleep Med. (2008) 4:487–504. doi: 10.5664/jcsm.27286 PMC2576317 18853708

[87] Wu JW Hussaini SA Bastille IM Rodriguez GA Mrejeru A Rilett K. Neuronal activity enhances tau propagation and tau pathology in *vivo* . Nat Neurosci. (2016) 19:1085–92. doi: 10.1038/nn.4328 PMC4961585 27322420

[88] Roehrs T Roth T. Drug-related sleep stage changes: functional significance and clinical relevance. Sleep Med Clin. (2010) 5:559–70. doi: 10.1016/j.jsmc.2010.08.002 PMC3041980 21344068

[89] Ciccone GK Holdcroft A. Drugs and sex differences: a review of drugs relating to anaesthesia. Br J Anaesth. (1999) 82:255–65. doi: 10.1093/bja/82.2.255 10365004

[90] Meador KJ. Cognitive outcomes and predictive factors in epilepsy. Neurology. (2002) 58:S21–6. doi: 10.1212/WNL.58.8_suppl_5.S21 11971129

[91] Beghi E Beghi M. Epilepsy, antiepileptic drugs and dementia. Curr Opin Neurol. (2020) 33:191–7. doi: 10.1097/WCO.0000000000000802 32073437

[92] Vossel K Ranasinghe KG Beagle AJ La A Ah Pook K Castro M. Effect of levetiracetam on cognition in patients with alzheimer disease with and without epileptiform activity: A randomized clinical trial. JAMA Neurol. (2021) 78:1345–54. doi: 10.1001/jamaneurol.2021.3310 PMC8477304 34570177

[93] Huang YH Pan MH Yang HI. The association between Gabapentin or Pregabalin use and the risk of dementia: an analysis of the National Health Insurance Research Database in Taiwan. Front Pharmacol. (2023) 14:1128601. doi: 10.3389/fphar.2023.1128601 37324474 PMC10266423

[94] Targa Dias Anastacio H Matosin N Ooi L. Neuronal hyperexcitability in Alzheimer's disease: what are the drivers behind this aberrant phenotype? Transl Psychiatry. (2022) 12:257. doi: 10.1038/s41398-022-02024-7 35732622 PMC9217953

[95] Hill SK Bishop JR Palumbo D Sweeney JA. Effect of second-generation antipsychotics on cognition: current issues and future challenges. Expert Rev Neurother. (2010) 10:43–57. doi: 10.1586/ern.09.143 20021320 PMC2879261

[96] Dong R Yuan L Yang Y Du XD Jia Q Dillon BA. Differential effects of different antipsychotic drugs on cognitive function in patients with chronic schizophrenia. Hum Psychopharmacol. (2020) 35:1–8. doi: 10.1002/hup.2754 32945023

[97] Albert N Randers L Allott K Jensen HD Melau M Hjorthoj C. Cognitive functioning following discontinuation of antipsychotic medication. A naturalistic sub-group analysis from the OPUS II trial. Psychol Med. (2019) 49:1138–47. doi: 10.1017/S0033291718001836 30058511

[98] Clissold M Crowe SF. Comparing the effect of the subcategories of atypical antipsychotic medications on cognition in schizophrenia using a meta-analytic approach. J Clin Exp Neuropsychol. (2019) 41:26–42. doi: 10.1080/13803395.2018.1488952 30025491

[99] Hasselmo ME. The role of acetylcholine in learning and memory. Curr Opin Neurobiol. (2006) 16:710–5. doi: 10.1016/j.conb.2006.09.002 PMC2659740 17011181

[100] Meneses A Liy-Salmeron G. Serotonin and emotion, learning and memory. Rev Neurosci. (2012) 23:543–53. doi: 10.1515/revneuro-2012-0060 23104855

[101] Hoekstra S Bartz-Johannessen C Sinkeviciute I Reitan SK Kroken RA Loberg EM. Sex differences in antipsychotic efficacy and side effects in schizophrenia spectrum disorder: results from the BeSt InTro study. NPJ Schizophr. (2021) 7:39. doi: 10.1038/s41537-021-00170-3 34408155 PMC8373883

[102] Seeman MV. The pharmacodynamics of antipsychotic drugs in women and men. Front Psychiatry. (2021) 12:650904. doi: 10.3389/fpsyt.2021.650904 33897500 PMC8062799

[103] Qizilbash N Gregson J Johnson ME Pearce N Douglas I Wing K. BMI and risk of dementia in two million people over two decades: a retrospective cohort study. Lancet Diabetes Endocrinol. (2015) 3:431–6. doi: 10.1016/S2213-8587(15)00033-9 25866264

[104] Sun Z Wang ZT Sun FR Shen XN Xu W Ma YH. Late-life obesity is a protective factor for prodromal Alzheimer's disease: a longitudinal study. Aging (Albany NY). (2020) 12:2005–17. doi: 10.18632/aging.102738 PMC7053604 31986486

[105] Kang SY Kim YJ Jang W Son KY Park HS Kim YS. Body mass index trajectories and the risk for Alzheimer's disease among older adults. Sci Rep. (2021) 11:3087. doi: 10.1038/s41598-021-82593-7 33542352 PMC7862316

[106] Zandi PP Carlson MC Plassman BL Welsh-Bohmer KA Mayer LS Steffens DC. Hormone replacement therapy and incidence of Alzheimer disease in older women: the Cache County Study. JAMA. (2002) 288:2123–9. doi: 10.1001/jama.288.17.2123 12413371

[107] Kim J Combs K Downs J Tillman III F. Medication adherence: the elephant in the room. US Pharm. (2018) 43 (1):30–4.

