# Supplementary figures and images for: Association between CNS-active drugs and risk of Alzheimer’s and age-related neurodegenerative diseases
Source: Front Psychiatry. 2024 Feb 29;15:1358568. doi: 10.3389/fpsyt.2024.1358568 (PMC10937406; doi:10.3389/fpsyt.2024.1358568)

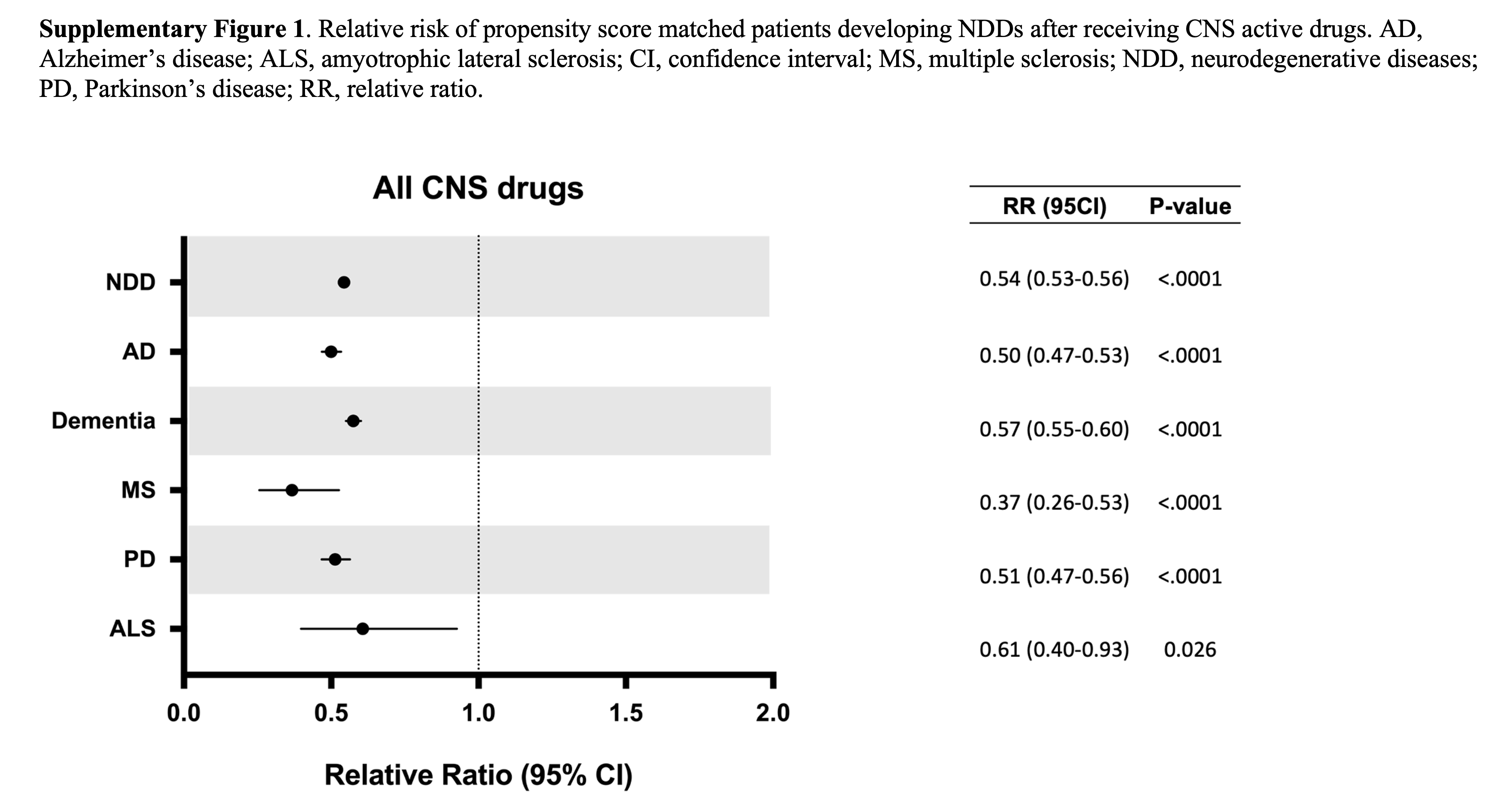

Supplement: Supplementary file 1 [file Image_1.png]

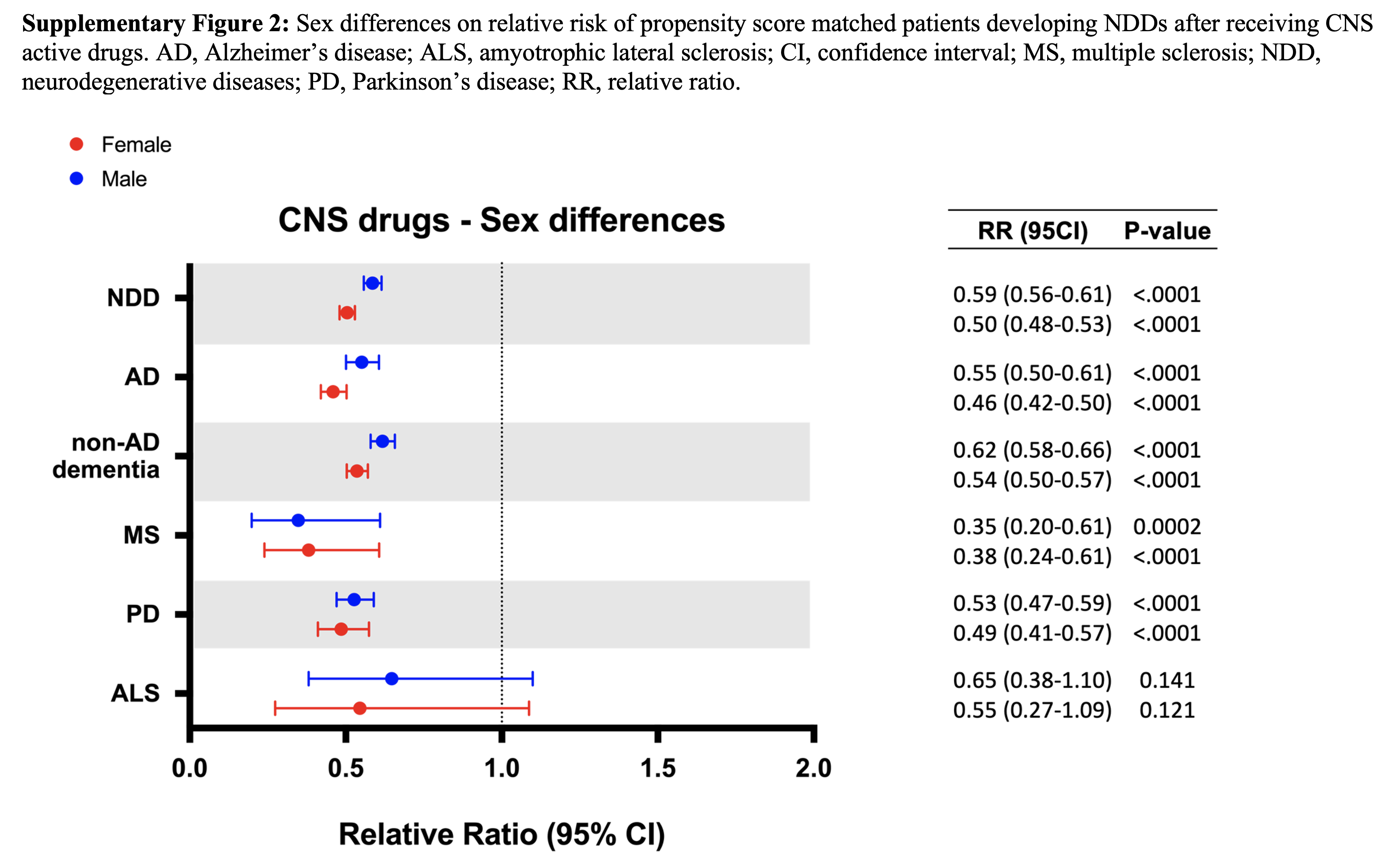

Supplement: Supplementary file 2 [file Image_2.png]
