# Supplementary material for: Association between CNS-active drugs and risk of Alzheimer’s and age-related neurodegenerative diseases
Source: Front Psychiatry. 2024 Feb 29;15:1358568. doi: 10.3389/fpsyt.2024.1358568 (PMC10937406; doi:10.3389/fpsyt.2024.1358568)
Supplement: Supplementary file 3 [file Table_1.docx]

**Supplementary Table 1:** List of drug codes used.

| **Drug** | **Code for Drugs** |
| --- | --- |
| Antidepressants | USC-64310, USC-64320, USC-64330, USC-64340, USC-64350, USC-64360 |
| Sedatives | USC-64610, USC-64690, GENERIC_DRUG-ZOLPIDEM_TARTRATE, GENERIC_DRUG-ESZOPICLONE, GENERIC_DRUG-ZALEPLON |
| Anticonvulsants | USC-67110, GENERIC_DRUG-PHENYTOIN, GENERIC_DRUG-PHENYTOIN_SODIUM_EXTENDED, GENERIC_DRUG-ETHOSUXIMIDE, GENERIC_DRUG-CARBAMAZEPINE, GENERIC_DRUG-OXCARBAZEPINE, GENERIC_DRUG-ESLICARBAZEPINE_ACETATE, GENERIC_DRUG-VALPROIC_ACID, GENERIC_DRUG-VALPROIC_ACID_(AS_SODIUM_SALT), GENERIC_DRUG-GABAPENTIN, GENERIC_DRUG-PREGABALIN, GENERIC_DRUG-FELBAMATE, GENERIC_DRUG-LAMOTRIGINE, GENERIC_DRUG-TOPIRAMATE, GENERIC_DRUG-TIAGABINE_HCL, GENERIC_DRUG-LEVETIRACETAM, GENERIC_DRUG-ZONISAMIDE |
| Antipsychotics | USC-64120, USC-64190 |
| Stimulants | GENERIC_DRUG-AMPHETAMINE, GENERIC_DRUG-AMPHETAMINE_SULFATE, GENERIC_DRUG-DEXTROAMPHETAMINE/AMPHETAMINE, GENERIC_DRUG-DEXTROAMPHETAMINE_SULFATE, GENERIC_DRUG-LISDEXAMFETAMINE_DIMESYLATE, GENERIC_DRUG-METHYLPHENIDATE, GENERIC_DRUG-DEXMETHYLPHENIDATE_HCL, GENERIC_DRUG-METHYLPHENIDATE_HCL |
| Tricyclics | USC-64310 |
| Monoamine oxidase inhibitors | USC-64320 |
| SSRI (Selective Serotonin Reuptake Inhibitors) | USC-64340 |
| SNRI (Serotonin/Norepinephrine Reuptake Inhibitors) | USC-64350 |
| SSRI/Serotonin Partial Agonists | USC-64360 |
| Benzodiazepines | USC-64610 |
| Z-drugs | GENERIC_DRUG-ZOLPIDEM_TARTRATE, GENERIC_DRUG-ESZOPICLONE, GENERIC_DRUG-ZALEPLON |
| First generation anticonvulsants | GENERIC_DRUG-CARBAMAZEPINE, GENERIC_DRUG-ETHOSUXIMIDE, GENERIC_DRUG-PHENOBARBITAL, GENERIC_DRUG-PHENYTOIN, GENERIC_DRUG-PRIMIDONE, GENERIC_DRUG-VALPROIC_ACID, GENERIC_DRUG-VALPROIC_ACID_(AS_SODIUM_SALT) |
| Second generation anticonvulsants | GENERIC_DRUG-FELBAMATE, GENERIC_DRUG-GABAPENTIN, GENERIC_DRUG-PREGABALIN, GENERIC_DRUG-LAMOTRIGINE, GENERIC_DRUG-LEVETIRACETAM, GENERIC_DRUG-OXCARBAZEPINE, GENERIC_DRUG-TIAGABINE_HCL, GENERIC_DRUG-TOPIRAMATE, GENERIC_DRUG-ZONISAMIDE |
| Typical antipsychotics | GENERIC_DRUG-HALOPERIDOL, GENERIC_DRUG-HALOPERIDOL_DECANOATE, GENERIC_DRUG-HALOPERIDOL_LACTATE, GENERIC_DRUG-CHLORPROMAZINE_HCL, GENERIC_DRUG-PERPHENAZINE |
| Atypical antipsychotics | GENERIC_DRUG-CLOZAPINE, GENERIC_DRUG-RISPERIDONE, GENERIC_DRUG-QUETIAPINE_FUMARATE, GENERIC_DRUG-OLANZAPINE, GENERIC_DRUG-ARIPIPRAZOLE |
| Stimulants | GENERIC_DRUG-AMPHETAMINE, GENERIC_DRUG-AMPHETAMINE_SULFATE, GENERIC_DRUG-DEXTROAMPHETAMINE/AMPHETAMINE, GENERIC_DRUG-DEXTROAMPHETAMINE_SULFATE, GENERIC_DRUG-LISDEXAMFETAMINE_DIMESYLATE, GENERIC_DRUG-METHYLPHENIDATE, GENERIC_DRUG-DEXMETHYLPHENIDATE_HCL, GENERIC_DRUG-METHYLPHENIDATE_HCL |
| Antidiabetics | GENERIC_DRUG-METFORMIN_HCL, GENERIC_DRUG-GLYBURIDE, GENERIC_DRUG-GLYBURIDE_MICRONIZED, GENERIC_DRUG-GLIMEPIRIDE, GENERIC_DRUG-GLIPIZIDE, GENERIC_DRUG-TOLAZAMIDE, GENERIC_DRUG-TOLBUTAMIDE, GENERIC_DRUG-CHLORPROPAMIDE, GENERIC_DRUG-PIOGLITAZONE_HCL, GENERIC_DRUG-ALOGLIPTIN_BENZ/PIOGLITAZONE, GENERIC_DRUG-PIOGLITAZONE_HCL/GLIMEPIRIDE, GENERIC_DRUG-PIOGLITAZONE_HCL/METFORMIN_HCL, GENERIC_DRUG-ROSIGLITAZONE/GLIMEPIRIDE, GENERIC_DRUG-ROSIGLITAZONE/METFORMIN_HCL, GENERIC_DRUG-ROSIGLITAZONE_MALEATE, GENERIC_DRUG-ALOGLIPTIN_BENZ/METFORMIN_HCL, GENERIC_DRUG-CANAGLIFLOZIN/METFORMIN_HCL, GENERIC_DRUG-DAPAGLIFLOZIN/METFORMIN_HCL, GENERIC_DRUG-EMPAGLIFLOZIN/METFORMIN_HCL, GENERIC_DRUG-ERTUGLIFLOZIN/METFORMIN, GENERIC_DRUG-GLIPIZIDE/METFORMIN_HCL, GENERIC_DRUG-GLYBURIDE/METFORMIN_HCL, GENERIC_DRUG-GLYBURIDE__MICRO/METFORMIN_HCL, GENERIC_DRUG-LINAGLIPTIN/METFORMIN_HCL, GENERIC_DRUG-METFORMIN/CAFF/AA7/HRB125/CHOL, GENERIC_DRUG-REPAGLINIDE/METFORMIN_HCL, GENERIC_DRUG-SAXAGLIPTIN_HCL/METFORMIN_HCL, GENERIC_DRUG-SITAGLIPTIN_PHOS/METFORMIN_HCL, GENERIC_DRUG-NATEGLINIDE, GENERIC_DRUG-REPAGLINIDE, GENERIC_DRUG-ACARBOSE, GENERIC_DRUG-MIGLITOL, GENERIC_DRUG-CANAGLIFLOZIN, GENERIC_DRUG-DAPAGLIFLOZIN/SAXAGLIPTIN_HCL, GENERIC_DRUG-DAPAGLIFLOZIN_PROPANEDIOL, GENERIC_DRUG-EMPAGLIFLOZIN, GENERIC_DRUG-EMPAGLIFLOZIN/LINAGLIPTIN, GENERIC_DRUG-ERTUGLIFLOZIN/SITAGLIPTIN, GENERIC_DRUG-ERTUGLIFLOZIN_PIDOLATE, GENERIC_DRUG-EXENATIDE, GENERIC_DRUG-EXENATIDE_MICROSPHERES, GENERIC_DRUG-LIXISENATIDE, GENERIC_DRUG-ALBIGLUTIDE, GENERIC_DRUG-DULAGLUTIDE, GENERIC_DRUG-LIRAGLUTIDE, GENERIC_DRUG-SEMAGLUTIDE, GENERIC_DRUG-TEDUGLUTIDE, GENERIC_DRUG-ALOGLIPTIN_BENZOATE, GENERIC_DRUG-LINAGLIPTIN, GENERIC_DRUG-SAXAGLIPTIN_HCL, GENERIC_DRUG-SITAGLIPTIN/SIMVASTATIN, GENERIC_DRUG-SITAGLIPTIN_PHOSPHATE, GENERIC_DRUG-HUM_INSULIN_NPH/REG_INSULIN_HM, GENERIC_DRUG-INFUSION_SET-INSULIN_PUMP_BODY, GENERIC_DRUG-INFUSION_SET_FOR_INSULIN_PUMP, GENERIC_DRUG-INSULIN_ADMIN._SUPPLIES, GENERIC_DRUG-INSULIN_ASPART, GENERIC_DRUG-INSULIN_ASPART_(NIACINAMIDE), GENERIC_DRUG-INSULIN_ASPART_PROT/INSULN_ASP, GENERIC_DRUG-INSULIN_ASPART_PROTAM_&_ASPART, GENERIC_DRUG-INSULIN_DEGLUDEC, GENERIC_DRUG-INSULIN_DEGLUDEC/LIRAGLUTIDE, GENERIC_DRUG-INSULIN_DETEMIR, GENERIC_DRUG-INSULIN_GLARGINE/LIXISENATIDE, GENERIC_DRUG-INSULIN_GLARGINE_HUM.REC.ANLOG, GENERIC_DRUG-INSULIN_GLULISINE, GENERIC_DRUG-INSULIN_ISOPHANE_NPH_BF-PK, GENERIC_DRUG-INSULIN_LISPRO, GENERIC_DRUG-INSULIN_LISPRO_PROTAMIN/LISPRO, GENERIC_DRUG-INSULIN_NPH_HUM/REG_INSULIN_HM, GENERIC_DRUG-INSULIN_NPH_HUMAN_ISOPHANE, GENERIC_DRUG-INSULIN_NPL/INSULIN_LISPRO, GENERIC_DRUG-INSULIN_PUMP/INFUS._SET/METER, GENERIC_DRUG-INSULIN_PUMP_CARTRIDGE, GENERIC_DRUG-INSULIN_PUMP_CONTROLLER, GENERIC_DRUG-INSULIN_PUMP_SYRINGE__1.8_ML, GENERIC_DRUG-INSULIN_PUMP_SYRINGE__3_ML, GENERIC_DRUG-INSULIN_REGULAR_BEEF-PORK, GENERIC_DRUG-INSULIN_REGULAR__HUMAN, GENERIC_DRUG-INSULIN_ULTRALENTE, GENERIC_DRUG-INSULIN_ZINC_BEEF-PORK, GENERIC_DRUG-INSULN_ASP_PRT/INSULIN_ASPART, GENERIC_DRUG-NEEDLELESS_ACCESS._DEV_INSULIN, GENERIC_DRUG-NEEDLES__INSULIN_DISP.__SAFETY, GENERIC_DRUG-NEEDLES__INSULIN_DISPOSABLE, GENERIC_DRUG-NPH__HUMAN_INSULIN_ISOPHANE, GENERIC_DRUG-SUB-Q_INSULIN_DEVICE__20_UNIT, GENERIC_DRUG-SUB-Q_INSULIN_DEVICE__30_UNIT, GENERIC_DRUG-SUB-Q_INSULIN_DEVICE__40_UNIT, GENERIC_DRUG-SUBCUTANEOUS_INSULIN_PUMP, GENERIC_DRUG-SUBQ_INSULIN_PUMP_GLUC.MON.SYS, GENERIC_DRUG-SYRINGE-NEEDLE_INSULIN_0.5_ML, GENERIC_DRUG-SYRINGE_&_NEEDLE_INSULIN_1_ML, GENERIC_DRUG-SYRINGE_AND_NEEDLE_INSULIN_1ML, GENERIC_DRUG-SYRINGE_INSULIN_NEEDLESS_1_ML, GENERIC_DRUG-SYRINGE_W-NDL__DISP.__INSULIN, GENERIC_DRUG-SYRINGE_WITH_NEEDLE__INSULIN, GENERIC_DRUG-SYR_NDL_INSULIN_1ML-SHARPS_BIN |
| Antihypertensives | GENERIC_DRUG-LISINOPRIL, GENERIC_DRUG-RAMIPRIL, GENERIC_DRUG-PERINDOPRIL_ERBUMINE, GENERIC_DRUG-QUINAPRIL_HCL, GENERIC_DRUG-CAPTOPRIL, GENERIC_DRUG-MOEXIPRIL_HCL, GENERIC_DRUG-TRANDOLAPRIL, GENERIC_DRUG-ENALAPRILAT_DIHYDRATE, GENERIC_DRUG-ENALAPRIL_MALEATE, GENERIC_DRUG-LISINOPRIL/HYDROCHLOROTHIAZIDE, GENERIC_DRUG-QUINAPRIL/HYDROCHLOROTHIAZIDE, GENERIC_DRUG-CAPTOPRIL/HYDROCHLOROTHIAZIDE, GENERIC_DRUG-MOEXIPRIL/HYDROCHLOROTHIAZIDE, GENERIC_DRUG-ENALAPRIL/HYDROCHLOROTHIAZIDE, GENERIC_DRUG-LOSARTAN_POTASSIUM, GENERIC_DRUG-VALSARTAN, GENERIC_DRUG-IRBESARTAN, GENERIC_DRUG-CANDESARTAN_CILEXETIL, GENERIC_DRUG-TELMISARTAN, GENERIC_DRUG-LOSARTAN/HYDROCHLOROTHIAZIDE, GENERIC_DRUG-VALSARTAN/HYDROCHLOROTHIAZIDE, GENERIC_DRUG-OLMESARTAN/HYDROCHLOROTHIAZIDE, GENERIC_DRUG-IRBESARTAN/HYDROCHLOROTHIAZIDE, GENERIC_DRUG-CANDESARTAN/HYDROCHLOROTHIAZID, GENERIC_DRUG-TELMISARTAN/HYDROCHLOROTHIAZID, GENERIC_DRUG-VERAPAMIL_HCL, GENERIC_DRUG-AMLODIPINE_BESYLATE, GENERIC_DRUG-DILTIAZEM_HCL, GENERIC_DRUG-FELODIPINE, GENERIC_DRUG-NICARDIPINE_HCL, GENERIC_DRUG-CHLOROTHIAZIDE, GENERIC_DRUG-CHLOROTHIAZIDE_SODIUM, GENERIC_DRUG-HYDROCHLOROTHIAZIDE, GENERIC_DRUG-CHLORTHALIDONE, GENERIC_DRUG-METOLAZONE |
| Anti-inflammatories | GENERIC_DRUG-PREDNISONE, GENERIC_DRUG-PREDNISONE_MICRONIZED, GENERIC_DRUG-GENTAMICIN_SULF/PREDNISOLONE, GENERIC_DRUG-METHYLPREDNISOLONE, GENERIC_DRUG-METHYLPREDNISOLONE/BUPIVACAINE, GENERIC_DRUG-METHYLPREDNISOLONE_ACETATE, GENERIC_DRUG-METHYLPREDNISOLONE_AC__MICRO, GENERIC_DRUG-METHYLPREDNISOLONE_SOD_SUCC, GENERIC_DRUG-METHYLPREDNISOLONE_SOD_SUCC/PF, GENERIC_DRUG-METHYLPREDNISOLONE__MICRONIZED, GENERIC_DRUG-PREDNISOLONE, GENERIC_DRUG-PREDNISOLONE_ACETATE, GENERIC_DRUG-PREDNISOLONE_ACETATE__MICRO, GENERIC_DRUG-PREDNISOLONE_SOD_PH/PEAK_FLOW, GENERIC_DRUG-PREDNISOLONE_SOD_PHOSPHATE, GENERIC_DRUG-PREDNISOLONE__MICRONIZED, GENERIC_DRUG-SULFACETAMIDE/PREDNISOLONE, GENERIC_DRUG-SULFACETAMIDE/PREDNISOLONE_SP, GENERIC_DRUG-CHLORPHEN/PHENYLEPH/IBUPROFEN, GENERIC_DRUG-CHLORPHEN/PSEUDOEPH/IBUPROFEN, GENERIC_DRUG-HYDROCODONE/IBUPROFEN, GENERIC_DRUG-IBUPROFEN, GENERIC_DRUG-IBUPROFEN/DIPHENHYDRAMINE_CIT, GENERIC_DRUG-IBUPROFEN/DIPHENHYDRAMINE_HCL, GENERIC_DRUG-IBUPROFEN/FAMOTIDINE, GENERIC_DRUG-IBUPROFEN/OXYCODONE_HCL, GENERIC_DRUG-IBUPROFEN/PHENYLEPHRINE_HCL, GENERIC_DRUG-IBUPROFEN/PSEUDOEPHEDRINE_HCL, GENERIC_DRUG-LANSOPRAZOLE/NAPROXEN, GENERIC_DRUG-NAPROXEN, GENERIC_DRUG-NAPROXEN/DIET._SUPP_11, GENERIC_DRUG-NAPROXEN/ESOMEPRAZOLE_MAG, GENERIC_DRUG-NAPROXEN_SOD/DIPHENHYDRAMINE, GENERIC_DRUG-NAPROXEN_SODIUM, GENERIC_DRUG-NAPROXEN_SODIUM/P-EPHED_HCL, GENERIC_DRUG-NAPROXEN_SODIUM/PSEUDOEPHEDRIN, GENERIC_DRUG-SUMATRIPTAN_SUCC/NAPROXEN_SOD, GENERIC_DRUG-INTERFERON_BETA-1A/ALBUMIN, GENERIC_DRUG-INTERFERON_BETA-1B, GENERIC_DRUG-INTERFERON_GAMMA-1B_RECOMB., GENERIC_DRUG-PEGINTERFERON_ALFA-2A, GENERIC_DRUG-PEGINTERFERON_ALFA-2B, GENERIC_DRUG-PEGINTERFERON_BETA-1A, GENERIC_DRUG-RIBAVIRIN/INTERFERON_A-2B, GENERIC_DRUG-FINGOLIMOD_HCL, GENERIC_DRUG-DIMETHYL_FUMARATE, GENERIC_DRUG-GLATIRAMER_ACETATE, GENERIC_DRUG-TERIFLUNOMIDE |
| SERMs | GENERIC_DRUG-100858, GENERIC_DRUG-100578, GENERIC_DRUG-105312, GENERIC_DRUG-101513, GENERIC_DRUG-104120, GENERIC_DRUG-105517, GENERIC_DRUG-103413 |
| ADTs | GENERIC_DRUG-LEUPROLIDE/NORETHINDRONE_ACET, GENERIC_DRUG-LEUPROLIDE_ACETATE, GENERIC_DRUG-FLUTAMIDE, GENERIC_DRUG-BICALUTAMIDE, GENERIC_DRUG-ABIRATERONE_ACETATE, GENERIC_DRUG-NILUTAMIDE, GENERIC_DRUG-ENZALUTAMIDE, GENERIC_DRUG-DEGARELIX_ACETATE, GENERIC_DRUG-GOSERELIN_ACETATE, GENERIC_DRUG-TRIPTORELIN_PAMOATE, GENERIC_DRUG-HISTRELIN_ACETATE, GENERIC_DRUG-KETOCONAZOLE, DRUG-LEUPROLIDE_ACETATE, DRUG-ELIGARD, DRUG-LUPRON, DRUG-LUPRON_DEPOT, DRUG-LUPRON_DEPOT-PED, DRUG-CASODEX, DRUG-ZYTIGA, DRUG-NILANDRON, DRUG-FIRMAGON, DRUG-ZOLADEX, DRUG-TRELSTAR, DRUG-VANTAS, DRUG-NIZORAL, DRUG-NIZORAL_A-D |
| Statins | GENERIC_DRUG-ATORVASTATIN_CALCIUM, GENERIC_DRUG-SIMVASTATIN, GENERIC_DRUG-ROSUVASTATIN_CALCIUM, GENERIC_DRUG-PRAVASTATIN_SODIUM, GENERIC_DRUG-LOVASTATIN, GENERIC_DRUG-FLUVASTATIN_SODIUM, GENERIC_DRUG-PITAVASTATIN_CALCIUM, DRUG-ATORVASTATIN_CALCIUM, DRUG-LOVASTATIN, DRUG-PRAVASTATIN_SODIUM, DRUG-SIMVASTATIN, DRUG-ROSUVASTATIN_CALCIUM, DRUG-FLUVASTATIN_SODIUM, DRUG-MEVACOR, DRUG-ALTOPREV, DRUG-LIPITOR, DRUG-PRAVACHOL, DRUG-ZOCOR, DRUG-CRESTOR, DRUG-LESCOL, DRUG-LESCOL_XL, DRUG-LIVALO |
| MHTs | DRUG-ALORA, DRUG-CENESTIN, DRUG-CLIMARA, DRUG-DELESTROGEN, DRUG-DIVIGEL, DRUG-ELESTRIN, DRUG-ENJUVIA, DRUG-ESTRACE, DRUG-ESTRADERM, DRUG-ESTRING, DRUG-EVAMIST, DRUG-FEMRING, DRUG-MENEST, DRUG-MENOSTAR, DRUG-MINIVELLE, DRUG-PREMARIN, DRUG-VIVELLE-DOT, DRUG-PROMETRIUM, DRUG-PROVERA, DRUG-ACTIVELLA, DRUG-ANGELIQ, DRUG-CLIMARA_PRO, DRUG-COMBIPATCH, DRUG-FEMHRT, DRUG-PREFEST, DRUG-PREMPRO |
