# Supplementary material for: Association between CNS-active drugs and risk of Alzheimer’s and age-related neurodegenerative diseases
Source: Front Psychiatry. 2024 Feb 29;15:1358568. doi: 10.3389/fpsyt.2024.1358568 (PMC10937406; doi:10.3389/fpsyt.2024.1358568)
Supplement: Supplementary file 4 [file Table_2.docx]

**Supplementary Table 2:** List of ICD-9 and ICD-10 diagnosis codes used

| **Diagnosis** | **ICD-9** | **ICD-10** |
| --- | --- | --- |
| Alzheimer’s disease | ICD-9-D-3310 | ICD-10-D-G300, ICD-10-D-G301, ICD-10-D-G308, ICD-10-D-G309 |
| Amyotrophic lateral sclerosis (ALS) | ICD-9-D-33520 | ICD-10-D-G1221 |
| Cardiovascular disease | ICD-9-D-4149, ICD-9-D-4280, ICD-9-D-4281, ICD-9-D-42820, ICD-9-D-42821, ICD-9-D-42822, ICD-9-D-42823, ICD-9-D-42830, ICD-9-D-42831, ICD-9-D-42832, ICD-9-42833, ICD-9-D-42840, ICD-9-D-42841, ICD-9-D-42842, ICD-9-D-42843, ICD-9-D-4289, ICD-9-D-4292 | ICD-10-D-I25700,ICD-10-D-I25708, ICD-10-D-I25709, ICD-10-D-I25710, ICD-10-D-I25719, ICD-10-D-I25720, ICD-10-D-I25729, ICD-10-D-I25739, ICD-10-D-I25798, ICD-10-D-I25799, ICD-10-D-I25810, ICD-10-D-I25811, ICD-10-D-I2582, ICD-10-D-I2583, ICD-10-D-I2584, ICD-10-D-I2589, ICD-10-D-I259, ICD-10-D-I501, ICD-10-D-I5043, ICD-10-D-I509 |
| COPD | ICD-9-D-49120, ICD-9-D-49121, ICD-9-D-49122, ICD-9-D-49320, ICD-9-D-49321, ICD-9-D-49322 | ICD-10-D-J440, ICD-10-D-J441, ICD-10-D-J449 |
| Chronic kidney disease | ICD-9-D-5851, ICD-9-D-5852, ICD-9-D-5853, ICD-9-D-5854, ICD-9-D-5855, ICD-9-D-5859 | ICD-10-D-N181, ICD-10-D-N182, ICD-10-D-N183, ICD-10-D-N184, ICD-10-D-N185, ICD-10-D-N189 |
| Congestive heart failure | ICD-9-D-7469, ICD-9-D-74689 | ICD-10-D-I5020, ICD-10-D-I5022, ICD-10-D-I5030, ICD-10-D-I5032, ICD-10-D-I5040, ICD-10-D-I5041, ICD-10-D-I5042 |
| Coronary artery disease | ICD-9-D-4110:ICD-9-D-4149 | ICD-10-D-I25:ICD-10-D-I259 |
| Diabetes | ICD-9-D-25000, ICD-9-D-25001, ICD-9-D-25002, ICD-9-D-25003 | ICD-10-D-E108, ICD-10-D-E109, ICD-10-D-E1169, ICD-10-D-E118, ICD-10-D-E119 |
| Hypercholesterolemia | ICD-9-D-2720 | ICD-10-D-E780, ICD-10-D-E7800 |
| Hypertension | ICD-9-D-4010, ICD-9-D-4011, ICD-9-D-4019 | ICD-10-D-I10, ICD-10-D-I110, ICD-10-D-I119 |
| Multiple sclerosis | ICD-9-D-340 | ICD-10-D-G35 |
| Neurosurgery or brain cancer | ICD-9-D-01320, ICD-9-D-01321, ICD-9-D-01322, ICD-9-D-01323, ICD-9-D-01325, ICD-9-D-01326, ICD-9-D-01330, ICD-9-D-01333, ICD-9-D-1917, ICD-9-D-1918, ICD-9-D-1919, ICD-9-D-1983, ICD-9-D-2250, ICD-9-D-2375, ICD-9-D-2396, ICD-9-D-3481, ICD-9-D-3484, ICD-9-D-34882, ICD-9-D-7422, ICD-9-D-V1085, ICD-9-D-V1241 | ICD-10-D-A066, ICD-10-D-A1781, ICD-10-D-A5482, ICD-10-D-B431, ICD-10-D-C717, ICD-10-D-C718, ICD-10-D-C719, ICD-10-D-C7931, ICD-10-D-D330, ICD-10-D-D331, ICD-10-D-D332, ICD-10-D-D430, ICD-10-D-D431, ICD-10-D-D432, ICD-10-D-D496, ICD-10-D-G931, ICD-10-D-G935, ICD-10-D-G9382, ICD-10-D-S06317A, ICD-10-D-S06317S, ICD-10-D-S06327A, ICD-10-D-S06337A, ICD-10-D-S06337S, ICD-10-D-S06377A, ICD-10-D-S06380A, ICD-10-D-S06380D, ICD-10-D-S06380S, ICD-10-D-S06381A, ICD-10-D-S06381D, ICD-10-D-S06382S, ICD-10-D-S06384A, ICD-10-D-S06385S, ICD-10-D-S06387A, ICD-10-D-S06387S, ICD-10-D-S06389A, ICD-10-D-S06389D, ICD-10-D-S06389S, ICD-10-D-Z85841, ICD-10-D-Z86011 |
| Non-Alzheimer’s dementia | ICD-9-D-2900, ICD-9-D-29010, ICD-9-D-29011, ICD-9-D-29012, ICD-9-D-29013, ICD-9-D-29020, ICD-9-D-29021, ICD-9-D-2903, ICD-9-D-29040, ICD-9-D-29041, ICD-9-D-29042, ICD-9-D-29043, ICD-9-D-29410, ICD-9-D-29411, ICD-9-D-29420, ICD-9-D-29421, ICD-9-D-33119, ICD-9-D-33182 | ICD-10-D-F0150, ICD-10-D-F0151, ICD-10-D-F0280, ICD-10-D-F0281, ICD-10-D-F0390, ICD-10-D-F0391, ICD-10-D-G3109, ICD-10-D-G3183 |
| Obesity | ICD-9-D-2780, ICD-9-D-27800, ICD-9-D-27801, ICD-9-D-27802, ICD-9-D-27803 | ICD-10-D-E660:ICD-10-D-E669 |
| Parkinson’s disease | ICD-9-D-332, ICD-9-D-3320 | ICD-10-D-G20, ICD-10-D-G214 |
| Stroke | ICD-9-D-430, ICD-9-D-431, ICD-9-D-432, ICD-9-D-4320, ICD-9-D-4321, ICD-9-D-4329, ICD-9-D-43300, ICD-9-D-4331, ICD-9-D-43310, ICD-9-D-43311, ICD-9-D-43320, ICD-9-D-43321, ICD-9-D-43330, ICD-9-D-43331, ICD-9-D-43381, ICD-9-D-43390, ICD-9-D-43391, ICD-9-D-43400, ICD-9-D-43401, ICD-9-D-43410, ICD-9-D-43411, ICD-9-D-43490, ICD-9-D-43491 | ICD-10-D-I6300, ICD-10-D-I63011, ICD-10-D-I63012, ICD-10-D-I63031, ICD-10-D-I63032, ICD-10-D-I6310, ICD-10-D-I63139, ICD-10-D-I63232, ICD-10-D-I63233, ICD-10-D-I63239, ICD-10-D-I63312, ICD-10-D-I6339, ICD-10-D-I6340, ICD-10-D-I63411, ICD-10-D-I63432, ICD-10-D-I6350, ICD-10-D-I63512, ICD-10-D-I63519, ICD-10-D-I63529, ICD-10-D-I6359, ICD-10-D-I638, ICD-10-D-I639 |
| Tobacco use | ICD-9-D-3051, ICD-9-D-98984, ICD-9-D-V1582 | ICD-10-D-F17220, ICD-10-D-F17221, ICD-10-D-F17223, ICD-10-D-F17228, ICD-10-D-F17229, ICD-10-D-F17290, ICD-10-D-F17291, ICD-10-D-F17293, ICD-10-D-F17298, ICD-10-D-F17299, ICD-10-D-Z720 |
