# Supplementary material for: Association between CNS-active drugs and risk of Alzheimer’s and age-related neurodegenerative diseases
Source: Front Psychiatry. 2024 Feb 29;15:1358568. doi: 10.3389/fpsyt.2024.1358568 (PMC10937406; doi:10.3389/fpsyt.2024.1358568)
Supplement: Supplementary file 5 [file Table_3.pdf]

Supplementary Table 3. List of CNS-active drug classes and subclasses included in the study, patient counts, and median adherence.

USC code	Drug Class	Drug name	Patient Number		Median Adherence (%)
**Antidepressants**					
64310	Tricyclics & Tetracyclics	Amitriptyline, Desipramine, Doxepin, Imipramine, Nortriptyline, Mirtazapine, Maprotiline	23,776 (8.31%)	88,826 (33.28%)	81.45%
64320	Monoamine Oxidase Inhibitors	Phenelzine, Selegiline, Tranylcypromine	85 (0.03%)		81.91%
64340	SSRI (Selective Serotonin Reuptake Inhibitors)	Fluoxetine, Sertraline, Paroxetine, Fluvoxamine, Citalopram, Escitalopram	55,994 (19.56%)		81.96%
64350	SNRI (Serotonin/Norepinephrine reuptake inhibitors)	Venlafaxine, Desmethylvenlafaxine, Milnacipran, Duloxetine	19,915 (6.96%)		86.71%
64360	SSRI/Serotonin Partial Agonists	Vilazodone	1,308 (0.46%)		89.55%
**Sedatives**					
64610	Benzodiazepines	Diazepam, Clonazepam, Lorazepam, Alprazolam, Triazolam	76,518 (26.73%)	103,715 (38.86%)	15.33%
	Z-drugs	Zolpidem, Eszopiclone, Zopiclone and Zaleplon	34,680 (12.11%)		43.34%
**Anticonvulsants**					
	First generation	Carbamazepine, Ethosuximide, Phenobarbital, Phenytoin, Primidone and Valproic Acid	3,924 (1.37%)	61,883 (23.19%)	86.33%
	Second generation	Felbamate, Gabapentin, Lamotrigine, Levetiracetam, Oxcarbazepine, Pregabaline, Tiagabine, Topiramate and Zonisamide	60,647 (21.18%)		71.24%
**Antipsychotics**					
	Typical (first generation)	Haloperidol, Chlorpromazine, Levomepromazine, Thioridazine, Perphenazine, Fluphenazine, Flupentixol, Trifluoperazine	1,893 (0.66%)	8,848 (3.32%)	72.73%
	Atypical (second generation)	Clozapine, Risperidone, Quetiapine, Olanzapine, Aripiprazole	7,545 (2.64%)		87.76%
**Stimulants**					
		Amphetamine, Dextroamphetamine, Lisdexamfetamine, Methylphenidate, Dexmethylphenidate	3,599 (1.35%)		64.29%
