# Supplementary material for: Association between CNS-active drugs and risk of Alzheimer’s and age-related neurodegenerative diseases
Source: Front Psychiatry. 2024 Feb 29;15:1358568. doi: 10.3389/fpsyt.2024.1358568 (PMC10937406; doi:10.3389/fpsyt.2024.1358568)
Supplement: Supplementary file 6 [file Table_4.pdf]

**Supplementary Table 4**: Relative risk of propensity score matched patients developing NDDs after receiving CNS active drugs. AD, Alzheimer's disease; ALS, amyotrophic lateral sclerosis; CI, confidence interval; MS, multiple sclerosis; NDD, neurodegenerative diseases; NNT, number needed to treat; PD, Parkinson's disease.

| | **All NDD Combined** | **AD** | **Non-AD dementia** | **MS** | **PD** | **ALS** |
|---|---|---|---|---|---|---|
| | | | | | | |
| | **Without exposure to CNS drugs** | | | | | |
| Patients not receiving CNS drugs | 8,902 | 2,673 | 5,208 | 109 | 1,280 | 56 |
| % | *5.76%* | *1.73%* | *3.37%* | *0.07%* | *0.83%* | *0.04%* |
| | **With exposure to CNS drugs** | | | | | |
| Patients receiving CNS drugs | 4,838 | 1,336 | 2,993 | 40 | 657 | 34 |
| % | *3.13%* | *0.86%* | *1.94%* | *0.03%* | *0.43%* | *0.02%* |
| Relative Risk | 0.54 | 0.50 | 0.57 | 0.37 | 0.51 | 0.61 |
| 95%CI | 0.53 to 0.56 | 0.47 to 0.53 | 0.55 to 0.60 | 0.26 to 0.53 | 0.47 to 0.56 | 0.40 to 0.93 |
| *p-value* | *<.0001* | *<.0001* | *<.0001* | *<.0001* | *<.0001* | *0.026* |
| NNT | 38.03 | 115.6 | 69.78 | 2,240 | 248.1 | 7,026 |
